# LCST phase behavior of benzo-21-crown-7 with different alkyl chains

**DOI:** 10.3762/bjoc.15.38

**Published:** 2019-02-14

**Authors:** Yan Deng, Xing Li, Qiao Zhang, Zheng Luo, Chengyou Han, Shengyi Dong

**Affiliations:** 1 College of Chemistry and Chemical Engineering, Hunan University, Changsha 410082, Hunan, P. R. China; 2 Department of Chemistry, College of science, China University of Petroleum (East China), Qingdao, 266580, P. R. China

**Keywords:** crown ethers, hydrophobic units, lower critical solution temperature, LCST, thermo-responsiveness, supramolecular chemistry

## Abstract

The introduction of hydrophobic units into crown ethers can dramatically decrease the critical transition temperature of LCST and realize macroscopic phase separation at low to moderate temperature and concentration. Minor modifications in the chemical structure of crown ethers (benzo-21-crown-7, B21C7s) can effectively control the thermo-responsive properties.

## Introduction

The introduction of stimuli-responsiveness into artificial materials is vital to design advanced functional materials [1–7]. An external thermal stimulus is often applied to supramolecular systems to realize reversible control over supramolecular self-assemblies [8–13]. In this context, lower critical solution temperature behavior, known as LCST behavior, is a type of thermo-responsiveness [14–16], which has gained much attention in supramolecular chemistry, because of its profound impact on the development of stimuli-sensitive supramolecular materials with controllable and/or programmable phase separation properties [17–19].

Though LCST behavior and LCST properties are always limited to polymeric systems [20–23], nowadays more and more efforts have been made to rationally design LCST systems involving low-molecular-weight monomers [24–26]. However, compared with polymeric LCST systems, only a limited number of smaller molecules-containing systems has been developed exhibiting LCST behavior and adjustable thermo-responsive properties. These include ionic liquids [27–30], macrocycles [31–34], and supramolecular pairs [26,35–38]. For example, the combination of macrocycles and supramolecular interactions can realize controlled release and product separation in complex supramolecular systems [35]. More options of small molecules with LCST properties not only give rise to more flexibility for LCST systems and thermo-responsive materials, but would be a great advantage for functionalization.

In our previous work, we have found that benzo-21-crown-7 (B21C7) and its derivatives exhibit typical LCST behavior in water, and that different substituted groups exert a great influence on the thermo-responsiveness [39]. However, highly concentrated solutions (>120 mg/mL) of B21C7s and elevated temperatures (>50 °C) are necessary to realize LCST phase separation, which significantly restricts an application in functional materials. In order to achieve LCST behavior of crown ethers at moderate concentrations and temperatures, two methods would be applied: combining multiple B21C7 units into a single molecule [40–41] or introducing hydrophobic units into B21C7 structures. For the first method, though the incorporation of B21C7s to polymeric backbones/cores can effectively lower the critical transition temperature, an accurate control of polymer/core structures is necessary but challenging. Because the balance between hydrophilicity and hydrophobicity is vital for an effective adjustment of phase behavior, herein we report a class of thermo-responsive B21C7s bearing different hydrophobic tails according to the second method and the results from investigations of the relationship between LCST properties and molecular structures.

## Results and Discussion

Two series of B21C7s, comprising carbamate-based linkers **3a**–**e** and urea-based linkers **5a**–**e**, were designed and synthesized, as shown in Scheme 1 and Scheme 2. All structures were confirmed by NMR spectroscopy and high-resolution mass spectrometry (details are provided in Supporting Information File 1, Figures S1–S34). Crown ethers with hydroxy groups (**2**) or amine groups (**4**) were prepared according to reported methods [42]. Hydrophobic alkyl chains with different lengths and branching were connected to the crown ether through condensation reactions between the amine groups and isocyanate units (yields, 44.6–76.5%), or between hydroxy groups and isocyanate units (yields, 57.4–84.9%), respectively. We also investigated the role of linkages in LCST behavior by the introduction of urea-based and carbamate-based linkers.

**Scheme 1:**
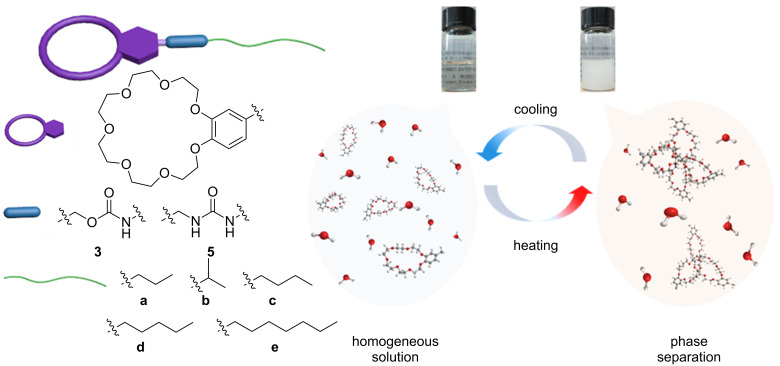
Chemical structures of **3a–e** and **5a–e**, and the cartoon representation of LCST behavior.

**Scheme 2:**
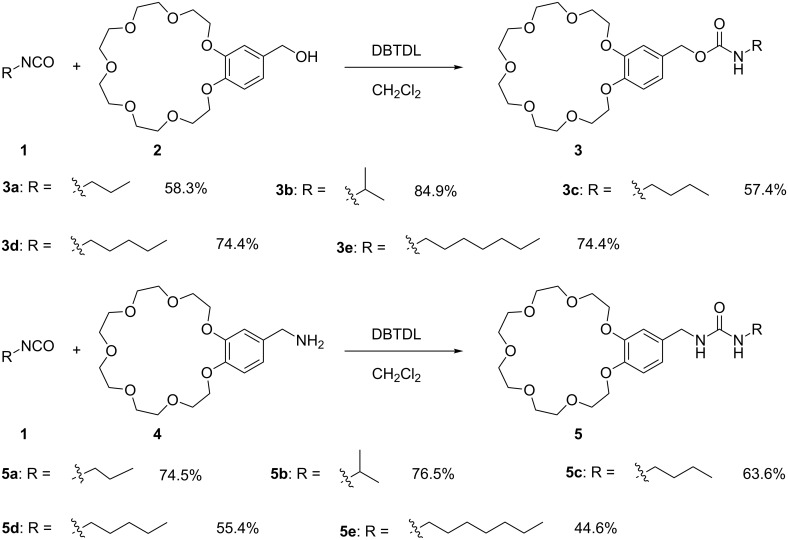
Synthetic routes and yields of **3a**–**e** and **5a**–**e**.

**Figure 1:**
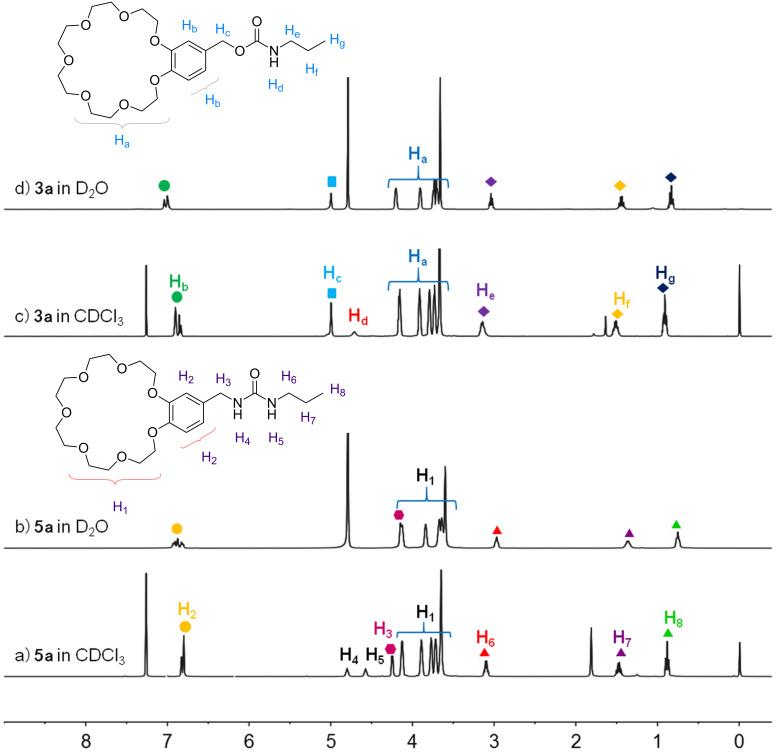
^1^H NMR spectra (room temperature) of (a) **5a** (5 mg/mL) in CDCl_3_; (b) **5a** (5 mg/mL) in D_2_O; (c) **3a** (5 mg/mL) in CDCl_3_; (d) **3a** (5 mg/mL) in D_2_O.

**Figure 2:**
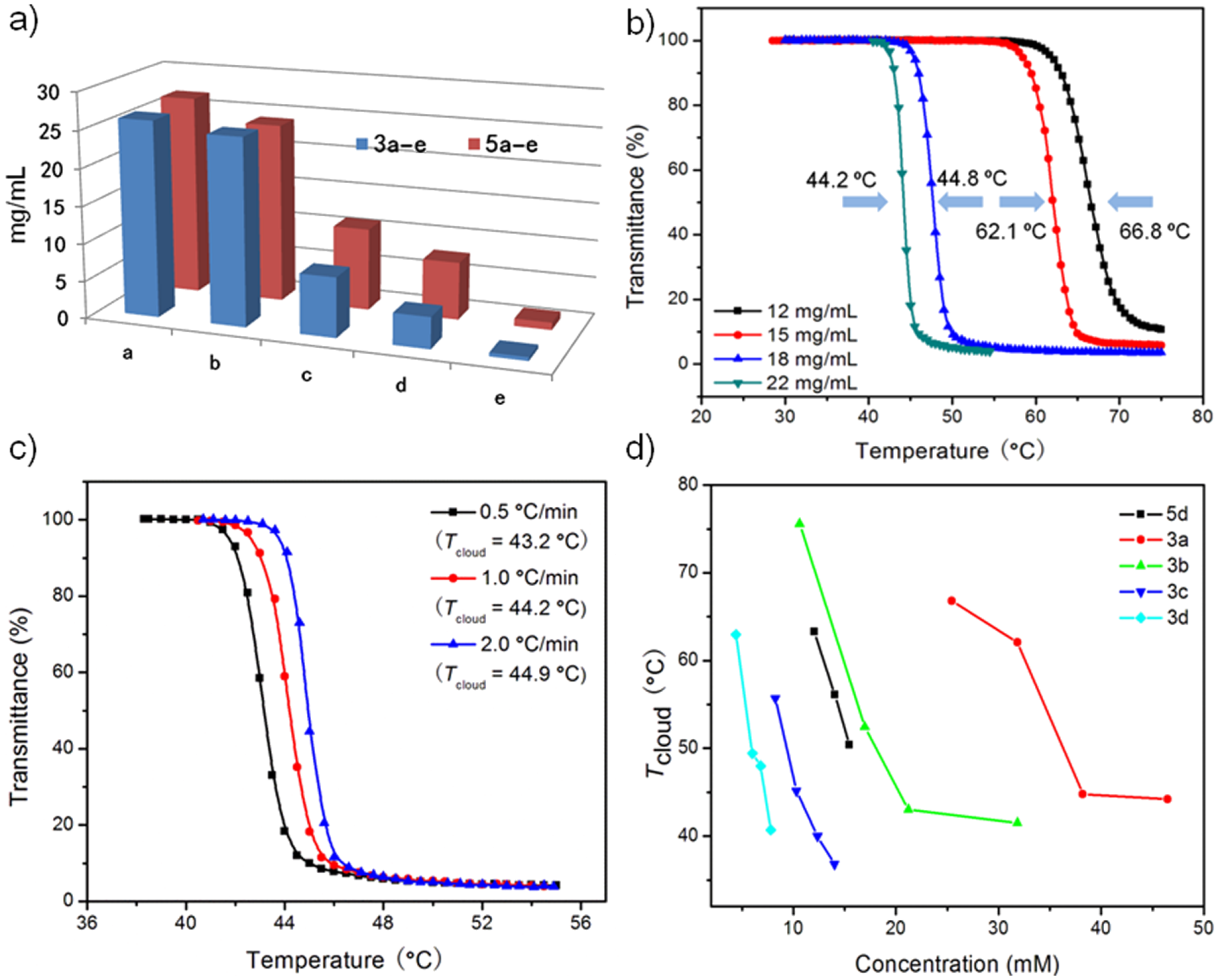
a) Water solubility of **3a**–**e** and **5a**–**e** at room temperature; b) concentration-dependent LCST behavior of **3a**; c) LCST behavior of **3a** at different heating rates; d) *T*_cloud_ of **3a**–**d** and **5d**.

The water solubility of the synthesized compounds **3a–e** and **5a–e** is crucial for LCST systems and thermo-responsiveness and was thus firstly studied. In our previous work [39], we have found that B21C7s substituted with cyano and amine functionalities showed high solubility (166.2 mg/mL and 197.8 mg/mL, respectively) in water at room temperature. ^1^H NMR of **3a–e** and **5a–e** in D_2_O were all successfully prepared (Figure 1 and further details are provided in Supporting Information File 1). However, compared with the two reported crown ethers, **3a–e** and **5a–e** all showed a relatively lower solubility in water. For example, at room temperature, the water solubility of **3a** and **5a** is 26.2 and 26.8 mg/mL, respectively. In addition, different alkyl chains exert a great effect on the water solubility. As shown in Figure 2a and Table S1 (Supporting Information File 1), it is quite obvious that the longer the alkyl chain is, the lower is the water solubility. When the alkyl chains contain seven CH_2_ units, the crown ethers become poorly soluble in water (the solubility is lower than 0.5 mg/mL for **3e**), indicating the hydrophobic effect of long alkyl chains. The nature of the linker unit is also closely related to the water solubility: in general, crown ethers with urea-based linkers show higher water solubility (**5a**–**e**), compared to crown ethers with carbamate-based linkers (**3a**–**e**). As amide groups are more hydrophilic compared with ester groups, it is reasonable that **5a**–**e** show stronger hydration effects and exhibit a larger water-accessible surface area, which means that **5a**–**e** are more soluble in water [39].

With the water solubility results in mind, we next turned to the studies of LCST properties of **3a**–**e** and **5a**–**e**. The results show that **3a**–**d** all displayed typical LCST behavior in water (Figure 2b–d, Figures S36–S39, Supporting Information File 1). For example, after heating the aqueous solution of **3a** (22 mg/mL) above 45 °C, an obvious transition from a transparent solution to a white turbid mixture was observed, indicating the macroscopic phase separation at elevated temperature (Scheme 1, insert photos). This transparent–turbid transition is fully reversible. Due to the poor solubility of **3e** in water, even a saturated solution of **3e** did not display LCST behavior (from UV–vis measurement, only about 12% decrease in transmittance was observed, when increasing the temperature to 70 °C), and no macroscopic phase separation was reached (Figure S40, Supporting Information File 1). Cloud temperature points (*T*_cloud_) recorded by UV–vis were further used to analyze the LCST behavior. All crown ethers **3a**–**d** showed obvious concentration-dependent *T*_cloud_ values (Figure 2d). For example, *T*_cloud_ of **3a** at 12 mg/mL (66.8 °C) is 22.6 °C higher than that of **3a** at 22 mg/mL (44.2 °C). It is demonstrated that, the longer the alkyl chain is the lower *T*_cloud_ becomes. We also found that the presence of a propyl substituent (**3a**) and an isopropyl group (**3b**) results in a quite different critical transition temperature, with *T*_cloud_ at 62.1 °C and 41.5 °C, respectively, when the concentration was kept the same for both samples (15 mg/mL). Consistent with reported crown ether systems [39], **3a**–**d** showed small hysteresis (around 2.0 °C) during heating and cooling cycles, indicating the chemical integrity during tests (Figure S36–S39, Supporting Information File 1). Different heating rates only exerted slight influence on the critical transition temperature (lower than 2.0 °C) and transition windows. For example, **3a** (22 mg/mL, Figure 2c) showed a 1.9 °C hysteresis during heating and cooling at the rate of 1.0 °C/min, while only caused a difference at around 1.7 °C at the heating rates from 0.5 °C/min to 2.0 °C/min.

**Figure 3:**
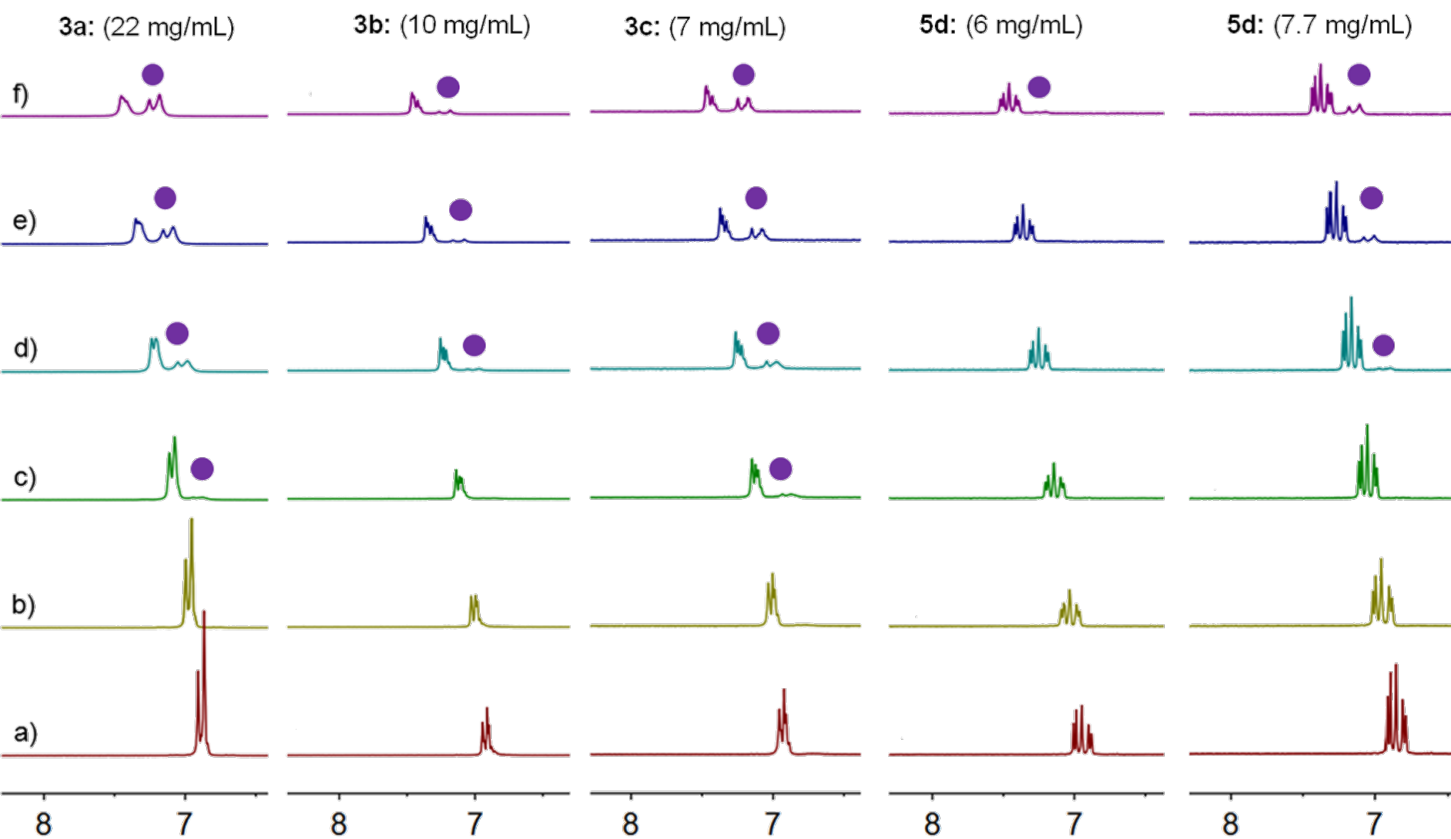
Temperature-dependent ^1^H NMR spectra (400 MHz, D_2_O) of **3a**–**c** and **5d**. Measurement at temperature a) 25 °C; b) 30 °C; c) 40 °C; d) 50 °C; e) 60 °C; f) 70 °C. Purple dots in spectra indicate the newly emergent peaks upon heating.

Further detailed information about LCST phase separation was obtained from temperature-dependent proton NMR measurements [33,39] (Figure 3 and Figure S42–S49, Supporting Information File 1). Taking **3a** (22 mg/mL) as an example, only small effects on the chemical shifts were observed when increasing the temperature from 25 to 40 °C, indicating that no thermo-induced aggregation occurred. However, when further increasing the temperature from 40 to 70 °C, new peaks gradually appeared, indicating the formation of a new species, which is consistent with the LCST behavior (*T*_cloud_ is 44.2 °C). With the increase of testing temperature, the intensity of the newly emergent peaks increased accordingly. Meanwhile, the normalized intensity of the NMR peaks of **3a** decreased as the temperature increased, indicating that not all aggregates can be detected by NMR measurements. For **3b**–**d**, not only changes in chemical shifts but also new peaks were found, when the temperature was higher than the compounds’ *T*_cloud_, which is similar to **3a**.

However, compared with thermo-responsiveness of **3a**–**d**, **5d** is the only crown ether among the urea-based series **5a**–**e**, showing LCST behavior in water. For the saturated solutions of **5a**, **5b**, **5c** and **5e**, no transparent–turbid transitions were observed even at temperatures close to the boiling point of water. Comparable to **3a–d**, crown ether **5d** also shows noticeable concentration-dependent properties above *T*_cloud_. The *T*_cloud_ of **5d** at 6.0 mg/mL, 7.0 mg/mL and 7.7 mg/mL, are 63.3, 56.1 and 50.4 °C, respectively, demonstrating that the higher crown ether concentration is, the lower the *T*_cloud_ value becomes. At concentrations of **5d** below 4.0 mg/mL, no LCST behavior was observed even at the boiling point of water. Different heating rates show minor effects on the LCST behavior of **5d** (0.7 °C difference in *T*_cloud_), as shown in Figure S35 (Supporting Information File 1). From the temperature-dependent NMR spectra (Figure 3, and Figure S42–S49 in Supporting Information File 1), it is apparent that both temperature and concentration are crucial for LCST behavior, which is consistent with the results obtained for **3a**–**d**. Spectra of **5d** at a higher concentration (7.7 mg/mL) show new peaks at a lower temperature (50 °C, Figure 3), compared with that of **5d** at a lower concentration (6 mg/mL, 70 °C). A relatively small hysteresis was observed (2.6 °C) during the heating and following cooling cycle. Based on these results, it is concluded that the nature of the linker unit is very important in realizing LCST behavior. Even minor changes in the linker (from NH to O) can result in remarkable differences of thermo-responsiveness. Once the hydrophilicity of linkage becomes more profound, the weak hydrogen bonding between water molecules and crown ethers are attenuated or destroyed, which will further prevent the aggregation of crown ethers and prohibit the occurrence of LCST.

By the analyses and comparisons of the LCST behaviors of **3a**–**e** and **5a**–**e**, the relationship between chemical structure and thermo-responsiveness can be disclosed: a) the introduction of hydrophobic units is an effective strategy to realize thermo-responsiveness at lower concentrations (4 to 20 mg/mL) and lower temperature (around 42 °C), compared with pure B21C7 (120 mg/mL, 55.1 °C). The *T*_cloud_ is more sensitive to the changes of concentrations with the decrease of water solubility; b) it is crucial to realize the balance between hydrophobicity and water solubility, wherein concentration and hydrophobicity are two key factors to control LCST behavior. Long alkyl chains not only change the hydrophobicity of the crown ethers, but also exert great effect on solubility; c) small modifications in the chemical structures can result in remarkable differences in thermo-responsiveness. The change from carbamate groups to urea groups leads to the quench of thermo-responsiveness of crown ethers (**5a**–**c**,**e**). Only one CH_2_ unit difference in alkyl chain length can lead to a 30 °C difference in *T*_cloud_ (**3a**, 66.8 °C, 12 mg/mL, Figure 2b; **3c**, 36.8 °C, 6.8 mg/mL, Figure S38, Supporting Information File 1).

## Conclusion

In conclusion, we here reported B21C7 derivatives with typical LCST behavior. The introduction of hydrophobic units into B21C7 structures can dramatically decrease the critical transition temperature and realize macroscopic phase separation at low to moderate temperature and concentration. Minor modifications in the chemical structure of B21C7 can effectively control thermo-responsive properties. Both linkers and tails are important for regulating the LCST phenomenon. The presence of hydrophobic tails has a greater influence on the solubility, but the nature of the linkers is more important for the LCST properties. Based on the analyses of the relationship between chemical structures and LCST properties, it was demonstrated that the balance between hydrophobicity and water solubility is crucial in designing LCST systems. These observations will be helpful in the design and functionalization of LCST systems. With the development of the LCST system, small compounds such as crown ethers can be used in two aspects, as they not only display LCST properties but also realize supramolecular control over thermo-responsiveness.

## Supporting Information

File 1 Experimental, characterization data, copies of spectra as well as solubility data and variable temperature UV–vis and NMR measurements.
