# Supplementary material for: LCST phase behavior of benzo-21-crown-7 with different alkyl chains
Source: Beilstein J Org Chem. 2019 Feb 14;15:437–44. doi: 10.3762/bjoc.15.38 (PMC6404474; doi:10.3762/bjoc.15.38)
Supplement: File 1 — Experimental, characterization data, copies of spectra as well as solubility data and variable temperature UV–vis and NMR measurements. [file Beilstein_J_Org_Chem-15-437-s001.pdf]

BEILSTEIN JOURNAL OF ORGANIC CHEMISTRY

Supporting Information

for

LCST phase behavior of benzo-21-crown-7 with different alkyl chains

Yan Deng, Xing Li, Qiao Zhang, Zheng Luo, Chengyou Han and Shengyi Dong

Beilstein J. Org. Chem. **2019,** *15,* 437–444. doi:10.3762/bjoc.15.38

Experimental, characterization data, copies of spectra as well as solubility data and variable temperature UV–vis and NMR measurements

License and Terms: This is a supporting information file under the terms of the Creative Commons Attribution License (http://creativecommons.org/licenses/by/4.0). Please note that the reuse, redistribution and reproduction in particular requires that the authors and source are credited.
The license is subject to the *Beilstein Journal of Organic Chemistry* terms and conditions: (https://www.beilstein-journals.org/bjoc)

Table of contents

1. Syntheses of **2, 4, 3a–e, 5a–e** S2

2. Solubility of **3a–e** and **5a–e**........ S22

3. Variable Temperature UV–vis of **5d**, **3a–e** S23

4. Variable Temperature ^{1}H NMR of **5d**, **3a–e** S29

1.Syntheses of 2, 4, 3a–e, 5a–e

Materials. All reagents were commercially available and used as supplied without further purification. Compounds **2** and **4** were synthesized according to reported methods [1,2].

Measurements. All variable temperature NMR, ^{1}H NMR and ^{13}C NMR spectra were recorded on a Bruker Ascend^{TM} 400 or 500 MHz spectrometer. The transmittance experiments were measured at 550 nm using a SHIMADZU 2600 UV–vis/NIR spectrometer with a temperature controllable system. Unless otherwise stated, samples were dissolved in Milli-Q water. High resolution mass spectrometry was performed on a Shimadzu Biotech AXIMA Performance instrument.

Synthesis of 2.

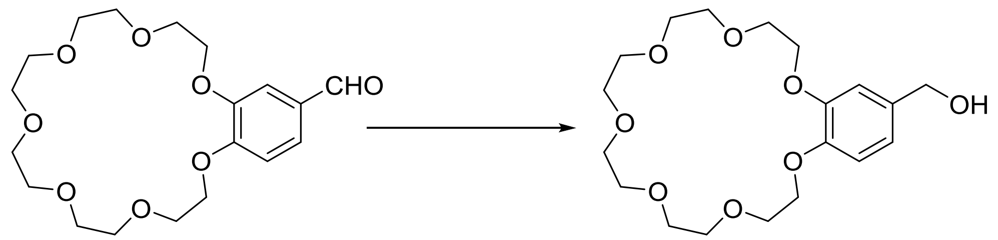

2

To a solution of **B21C7-CHO** (0.9 g, 2.3 mmol) in THF (50 mL) was added slowly $LiAlH_4$ (0.3 g, 6.9 mmol) in a three-necked flask under N_2 at 0 °C. After the mixture was refluxed overnight, $Na_2SO_4 \cdot 10H_2O$ was added to the mixture until there were no more bubbles generated. Then the mixture was filtered and THF was removed followed by extraction with CH_2Cl_2 three times. The organic phases were combined, washed with water, dried over Na_2SO_4. After filtration and solvent evaporation, product **2** was obtained as pale oil (0.74 g, 83.4%).

Synthesis of 4.

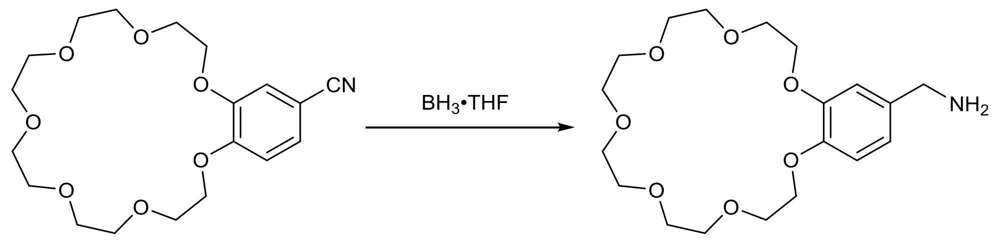

4

B21C7-CN (2.55 g, 6.67 mmol) was added to 1 M borane-tetrahydrofuran complex (66.9 mL, 66.9 mmol) at 0 °C. The solution was stirred for 30 min at 0 °C, and heated to reflux for 20 h. The reaction mixture was cooled to 0 °C, and CH_3OH (30 mL) was added dropwise. Hydrochloric acid (1 mL, 37% in water) was added slowly, the mixture stirred for 1 h and subsequently evaporated to dryness under reduced pressure. Trimethyl borate was removed by three subsequent coevaporations with methanol. Sodium hydroxide solution (80 mL, 1 M in water) was added to the viscous liquid, followed by extraction with CH_2Cl_2. The combined

organic layers were dried over anhydrous Na_2SO_4, filtered, and the solvent was evaporated on a rotary evaporator yielding a yellow oil (1.86 g, 74.5%)

Syntheses of 3a–e and 5a–e. Dibutyltin dilaurate (DBTDL) (1 drop) was added to the mixture of **1** (0.25 g of **1a**, 0.25 g of **1b**, 0.30 g of **1c**, 0.34 g of **1d** or 0.42 g of **1e**, 3.0 mmol) and **2** (0.38 g, 1.0 mmol) or **4** (0.38 g, 1.0 mmol) in dichloromethane. The reaction mixture was stirred at room temperature for one day. Then, water was added to quench the reaction, and the organic phase was separated, concentrated and the residue was subjected by column chromatography (10:1, CH_2Cl_2/MeOH, *v*:*v*) to give **3** or **5** as white solids.

3a (0.27 g, 58.3%): ^1H NMR (400 MHz, D_2O, room temperature): δ 7.04 (s, 1H), 6.99 (d, J = 4.0 Hz, 2H), 5.00 (s, 2H), 4.20 (s, 4H), 3.91 (s, 4H), 3.74 (d, J = 8.0 Hz, 4H), 3.70 (d, J = 8.0 Hz, 4H), 3.66 (s, 8H), 3.04 (t, J = 8.0 Hz, 2H), 1.45–1.42 (m, 2H), 0.83 (t, J = 8.0 Hz, 3H). ^1H NMR (400 MHz, $CDCl_3$, room temperature): δ 6.88 (s, 2H), 6.83 (s, 1H), 4.97 (s, 2H), 4.78 (s, 1H), 4.13 (s, 4H), 3.89 (s, 4H), 3.77 (s, 4H), 3.71 (s, 4H), 3.65 (s, 8H), 3.12 (d, J = 8.0 Hz, 2H), 1.50–1.46 (m, 2H), 0.88 (t, J = 8.0 Hz, 3H). ^{13}C NMR (125 MHz, $CDCl_3$, room temperature): δ 156.46, 148.85, 129.77, 121.60, 114.45, 113.92, 71.16, 71.11, 71.02, 70.54, 69.73, 69.32, 69.29, 66.51, 42.78, 23.19, 11.23. LRESIMS: *m*/*z* 493.67 $[M + Na]^+$, 509.66 $[M + K]^+$. HRESIMS: *m*/*z* calcd for $[M + K]^+$ $C_{23}H_{37}KNO_9$, 510.2105; found 510.2127, error 4.30 ppm.

3b (0.40 g, 84.9%): ^1H NMR (400 MHz, D_2O, room temperature): δ 7.04 (s, 1H), 7.00 (d, J = 4.0 Hz, 2H), 4.99 (s, 2H), 4.20 (d, J = 4.0 Hz, 4H), 3.91 (d, J = 4.0 Hz, 4H), 3.74 (d, J = 4.0 Hz, 4H), 3.70 (d, J = 4.0 Hz, 4H), 3.66 (s, 8H), 3.61 (d, J = 4.0 Hz, 1H), 1.08 (t, J = 8.0 Hz, 6H). ^1H NMR (400 MHz, $CDCl_3$, room temperature): δ 6.90 (s, 2H), 6.85 (s, 1H), 4.98 (s, 2H), 4.55 (s, 1H), 4.15 (s, 4H), 3.91 (s, 4H), 3.85 (s, 1H), 3.79 (s, 4H), 3.73 (s, 4H), 3.67 (s, 8H), 1.14 (d, J = 4.0 Hz, 6H). ^{13}C NMR (125 MHz, $CDCl_3$, room temperature): δ 155.57, 148.86, 148.83, 129.77, 121.58, 114.45, 113.94, 71.17, 71.12, 71.03, 70.88, 70.64, 70.54, 69.73, 69.34, 69.30, 66.39, 43.10, 23.03. LRESIMS: *m*/*z* 493.67 $[M + Na]^+$, 509.66 $[M + K]^+$. HRESIMS: *m*/*z* calcd for $[M + K]^+$ $C_{23}H_{37}KNO_9$, 510.2105; found 510.2095, error -1.95 ppm.

3c (0.27 g, 57.4%): ^1H NMR (400 MHz, D_2O, room temperature): δ 7.04 (s, 1H), 7.00 (d, J = 4.0 Hz, 1H), 5.00 (s, 2H), 4.20 (s, 4H), 3.91 (s, 4H), 3.74 (d, J = 4.0 Hz, 4H), 3.70 (d, J = 4.0 Hz, 4H), 3.67 (s, 8H), 3.08 (t, J = 8.0 Hz, 2H), 1.45–1.38 (m, 2H), 1.29–1.23 (m, 2H), 0.84 (t, J = 8.0 Hz, 3H). ^1H NMR (400 MHz, $CDCl_3$, room temperature): δ 6.90 (s, 2H), 6.85 (s, 1H), 4.99 (s, 2H), 4.69 (s, 1H), 4.15 (s, 4H), 3.91 (s, 4H), 3.79 (s, 4H), 3.73 (s, 4H), 3.67 (s, 8H), 3.18 (d, J = 8.0 Hz, 2H), 1.49–1.45 (m, 2H), 1.36–1.31 (m, 2H), 0.91 (t, J = 8.0 Hz, 3H). ^{13}C NMR (125 MHz, $CDCl_3$, room temperature): δ 156.44, 148.85, 129.77, 121.59, 114.44, 113.91, 71.15, 71.10, 71.01, 70.54, 69.72, 69.31, 69.27, 66.49, 40.77, 32.02, 19.88, 13.74. LRESIMS: *m*/*z* 507.71 $[M + Na]^+$, 523.70 $[M + K]^+$. HRESIMS: *m*/*z* calcd for $[M + K]^+$ $C_{24}H_{39}KNO_9$, 524.2262; found 524.2284, error 4.19 ppm.

3d (0.37 g, 74.4%): ^1H NMR (400 MHz, D_2O, room temperature): δ 7.06 (s, 1H), 7.02 (d, J = 8.0 Hz, 2H), 5.02 (s, 2H), 4.23 (s, 4H), 3.92 (s, 4H), 3.75 (s, 4H), 3.72 (s, 4H), 3.67 (s, 8H), 3.08 (t, J = 8.0 Hz, 2H), 1.47–1.41 (m, 2H), 1.23 (s, 4H), 0.82 (t, J = 8.0 Hz, 3H). ^1H NMR (400 MHz, $CDCl_3$, room temperature): δ 6.90 (s, 2H), 6.86 (s, 1H), 5.00 (s, 2H), 4.68 (s, 1H), 4.16 (s, 4H), 3.91 (s, 4H), 3.79 (s, 4H), 3.73 (s, 4H), 3.67 (s, 8H), 3.17 (d, J = 4.0 Hz, 2H), 1.49 (s, 2H), 1.30 (s, 4H), 0.88 (d, J = 8.0 Hz, 3H). ^{13}C NMR (125 MHz, $CDCl_3$, room temperature): δ 156.43, 148.86, 129.78, 121.60, 114.47, 113.93, 71.16, 71.11, 71.02, 70.64, 70.54, 69.73, 69.33, 69.29, 66.51, 41.07, 29.65, 28.89, 22.34, 14.01. LRESIMS: *m*/*z* 521.75

[M + Na]$^+$, 537.68 [M + K]$^+$. HRESIMS: *m*/*z* calcd for [M + K]$^+$ $C_{25}H_{41}KNO_9$, 538.2418; found 538.2442, error 4.45 ppm.

3e (0.39 g, 74.4%): ^{1}H NMR (400 MHz, $CDCl_3$, room temperature): δ 6.90 (s, 2H), 6.85 (s, 1H), 4.99 (s, 2H), 4.68 (s, 1H), 4.15 (s, 4H), 3.91 (s, 4H), 3.79 (s, 4H), 3.73 (s, 4H), 3.67 (s, 8H), 3.17 (d, *J* = 8.0 Hz, 2H), 1.47 (d, *J* = 8.0 Hz, 2H), 1.26 (d, *J* = 8.0 Hz, 8H), 0.87 (t, *J* = 8.0 Hz, 3H). ^{13}C NMR (125 MHz, $CDCl_3$, room temperature): δ 156.41, 148.89, 129.80, 121.60, 114.52, 113.99, 71.16, 71.12, 71.03, 70.55, 69.74, 69.36, 69.32, 66.50, 41.10, 31.73, 29.97, 28.93, 26.70, 22.57, 14.07. LRESIMS: 549.77 [M + Na]$^+$, 565.70 [M + K]$^+$. HRESIMS: *m*/*z* calcd for [M + K]$^+$ $C_{27}H_{45}KNO_9$, 566.2731; found 566.2750, error 3.35 ppm.

5a (0.35 g, 74.5%): ^{1}H NMR (400 MHz, D_2O, room temperature): δ 6.92 (d, *J* = 8.0 Hz, 1H), 6.87 (s, 1H), 6.82 (d, *J* = 8.0 Hz, 1H), 4.14 (d, *J* = 8.0 Hz, 6H), 3.84 (s, 4H), 3.67 (s, 4H), 3.64 (s, 4H), 3.60 (s, 8H), 2.96 (d, *J* = 8.0 Hz, 2H), 1.36 (d, *J* = 8.0 Hz, 2H), 0.75 (t, *J* = 8.0 Hz, 3H). ^{1}H NMR (400 MHz, $CDCl_3$, room temperature) δ 6.83 (s, 1H), 6.80 (s, 2H), 4.80 (s, 1H), 4.57 (s, 1H), 4.25 (d, *J* = 4.0 Hz, 2H), 4.13 (d, *J* = 4.0 Hz, 4H), 3.89 (s, 4H), 3.77 (d, *J* = 4.0 Hz, 4H), 3.72 (s, 4H), 3.65 (s, 8H), 3.12–3.07 (m, 2H), 1.50–1.45 (m, 2H), 0.88 (t, *J* = 8.0 Hz, 3H). ^{13}C NMR (125 MHz, $CDCl_3$, room temperature): δ 158.54, 149.01, 147.93, 133.16, 120.31, 114.31, 113.61, 71.02, 70.95, 70.50, 70.46, 69.79, 69.40, 69.17, 44.12, 42.18, 23.48, 11.37. LRESIMS: *m*/*z* 470.67 [M + H]$^+$, 492.66 [M + Na]$^+$, 508.67 [M + K]$^+$. HRESIMS: *m*/*z* calcd for [M + Na]$^+$ $C_{23}H_{38}N_2NaO_8$, 493.2526; found 493.2536, error 2.02 ppm.

5b (0.36 g, 76.5%): ^{1}H NMR (400 MHz, D_2O, room temperature): δ 6.92 (d, *J* = 8.0 Hz, 1H), 6.87 (s, 1H), 6.81 (d, *J* = 8.0 Hz, 1H), 4.14 (s, 6H), 3.84 (s, 4H), 3.67 (s, 4H), 3.64 (s, 4H), 3.60 (s, 8H), 1.01 (d, *J* = 4.0 Hz, 6H). ^{1}H NMR (400 MHz, $CDCl_3$, room temperature): δ 6.84 (s, 1H), 6.80 (s, 2H), 4.69 (s, 1H), 4.34 (s, 1H), 4.24 (d, *J* = 8.0 Hz, 2H), 4.14 (s, 4H), 3.89 (s, 4H), 3.85–3.82 (m, 1H), 3.77 (d, *J* = 4.0 Hz, 4H), 3.72 (s, 4H), 3.65 (s, 8H), 1.11 (d, *J* = 4.0 Hz, 6H). ^{13}C NMR (125 MHz, $CDCl_3$, room temperature): δ 157.84, 148.96, 147.88, 133.06, 120.34, 114.31, 113.64, 71.02, 70.98, 70.92, 70.47, 70.43, 69.78, 69.37, 69.14, 44.01, 42.05, 23.45. LRESIMS: m/z 470.67 [M + H]$^+$, 492.66 [M + Na]$^+$, 508.61 [M + K]$^+$. HRESIMS: *m*/*z* calcd for [M + K]$^+$ $C_{23}H_{38}KN_2O_9$, 509.2265; found 509.2306, error 8.05 ppm.

5c (0.31 g, 63.6%): ^{1}H NMR (400 MHz, D_2O, room temperature): δ 6.92 (d, *J* = 8.0 Hz, 1H), 6.87 (s, 1H), 6.81 (d, *J* = 8.0Hz, 1H), 4.13 (d, *J* = 8.0 Hz, 6H), 3.84 (s, 4H), 3.67 (s, 4H), 3.64 (s, 4H), 3.60 (s, 8H), 3.00 (s, 2H), 1.32 (s, 2H), 1.15 (d, *J* = 8.0 Hz, 2H), 0.77 (t, *J* = 8.0 Hz, 3H). ^{1}H NMR (400 MHz, $CDCl_3$, room temperature): δ 6.83 (s, 1H), 6.80 (s, 2H), 4.73 (s, 1H), 4.47 (s, 1H), 4.25 (d, *J* = 8.0 Hz, 2H), 4.13 (s, 4H), 3.90 (s, 4H), 3.77 (d, *J* = 4.0 Hz, 4H), 3.72 (s, 4H), 3.65 (s, 8H), 3.16–3.11 (m, 2H), 1.46–1.42 (m, 2H), 1.34–1.28 (m, 2H), 0.89 (t, *J* = 8.0 Hz, 3H). ^{13}C NMR (125 MHz, $CDCl_3$, room temperature): δ 158.55, 148.99, 147.89, 133.05, 120.29, 114.32, 113.58, 71.01, 70.94, 70.49, 70.44, 69.79, 69.40, 69.15, 44.07, 40.13, 32.40, 20.04, 13.84. LRESIMS: *m*/*z* 484.65 [M + H]$^+$, 506.64 [M + Na]$^+$, 522.66 [M + K]$^+$. HRESIMS: *m*/*z* calcd for [M + K]$^+$ $C_{24}H_{40}KN_2O_8$, 523.2422; found 523.2430, error 1.52 ppm.

5d (0.28 g, 55.4%): ^{1}H NMR (400 MHz, D_2O, room temperature): δ 6.90 (d, *J* = 8.0 Hz, 1H), 6.86 (s, 1H), 6.81 (d, *J* = 8.0 Hz, 1H), 4.12 (d, *J* = 4.0 Hz, 6H), 3.83 (s, 4H), 3.67 (s, 4H), 3.64 (s, 4H), 3.60 (s, 8H), 3.00 (s, 2H), 1.33 (s, 2H), 1.15–1.11 (m, 4H), 0.74 (s, 3H). ^{1}H NMR (400 MHz, $CDCl_3$, room temperature): δ 6.83 (s, 1H), 6.80 (s, 2H), 4.76 (s, 1H), 4.51 (s, 1H), 4.25 (d, *J* = 4.0 Hz, 2H), 4.13 (s, 4H), 3.89 (s, 4H), 3.77 (s, 4H), 3.72 (d, *J* = 4.0 Hz, 4H), 3.65

(s, 8H), 3.15–3.10 (m, 2H), 1.47–1.44 (m, 2H), 1.31–1.28 (m, 4H), 0.87 (t, J = 8.0 Hz, 3H). ^{13}C NMR (125 MHz, $CDCl_3$, room temperature): δ 158.45, 149.02, 147.96, 132.89, 120.32, 114.29, 113.61, 71.05, 71.01, 70.94, 70.50, 70.46, 69.79, 69.37, 69.16, 44.17, 40.49, 29.95, 29.05, 22.40, 14.04. LRESIMS: m/z 498.70 $[M + H]^+$, 520.69 $[M + Na]^+$, 536.70 $[M + K]^+$. HRESIMS: m/z calcd for $[M + K]^+$ $C_{25}H_{42}KN_2O_8$, 537.2578; found 537.2587, error 1.70 ppm.

5e (0.23 g, 44.6%): ^{1}H NMR (400 MHz, $CDCl_3$, room temperature): δ 6.85 (s, 1H), 6.81 (s, 2H), 4.62 (s, 1H), 4.37 (s, 1H), 4.27 (d, J = 4.0 Hz, 2H), 4.14 (s, 4H), 3.90 (s, 4H), 3.78 (s, 4H), 3.73 (s, 4H), 3.66 (s, 8H), 3.16–3.11 (m, 2H), 1.46 (s, 2H), 1.27 (s, 8H), 0.87 (t, J = 8.0 Hz, 3H). ^{13}C NMR (125 MHz, $CDCl_3$, room temperature): δ 158.40, 149.05, 148.02, 132.81, 120.33 , 114.31, 113.65, 71.09, 70.05, 70.97, 70.52, 70.48, 69.80, 69.40, 69.20, 44.22, 40.56, 31.79, 30.27, 29.04, 26.87, 22.61, 14.10. LRESIMS: m/z 526.73 $[M + H]^+$, 548.72 $[M + Na]^+$, 564.73 $[M + K]^+$. HRESIMS: m/z calcd for $[M + K]^+$ $C_{27}H_{46}KN_2O_8$, 565.2891; found 565.2908, error 3.01 ppm.

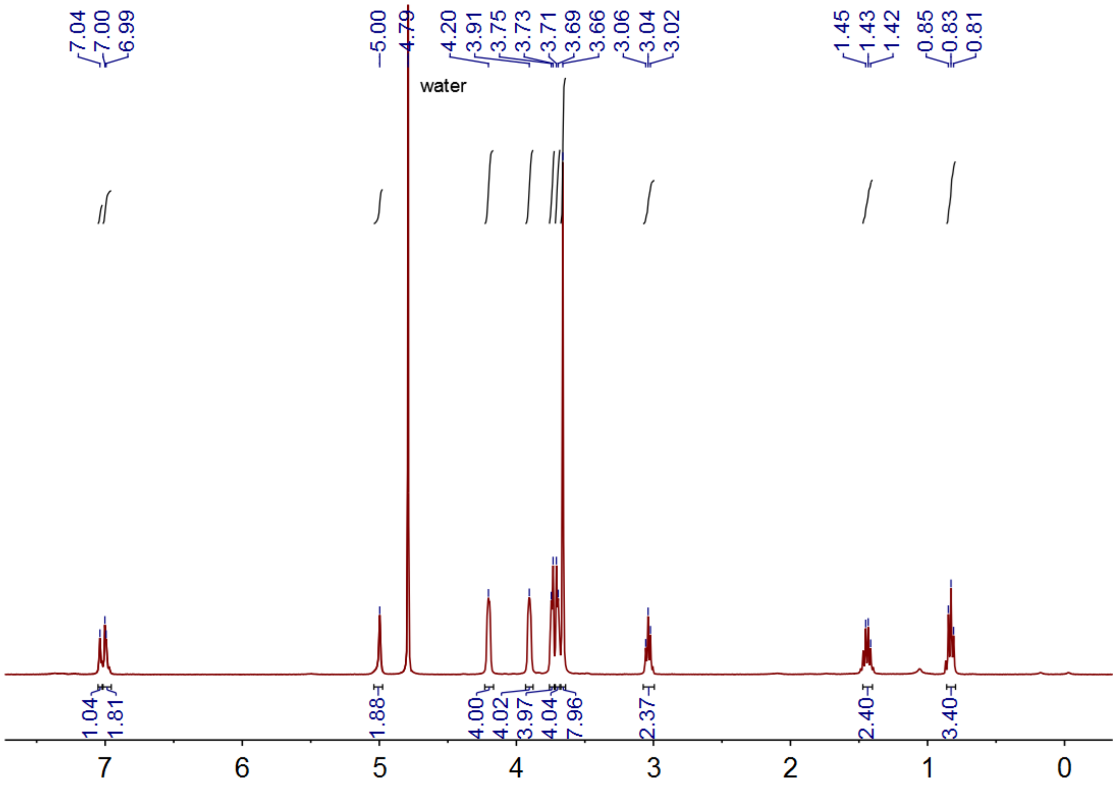

Figure S1: ^{1}H NMR spectrum (400 MHz, D_2O, room temperature) of **3a**

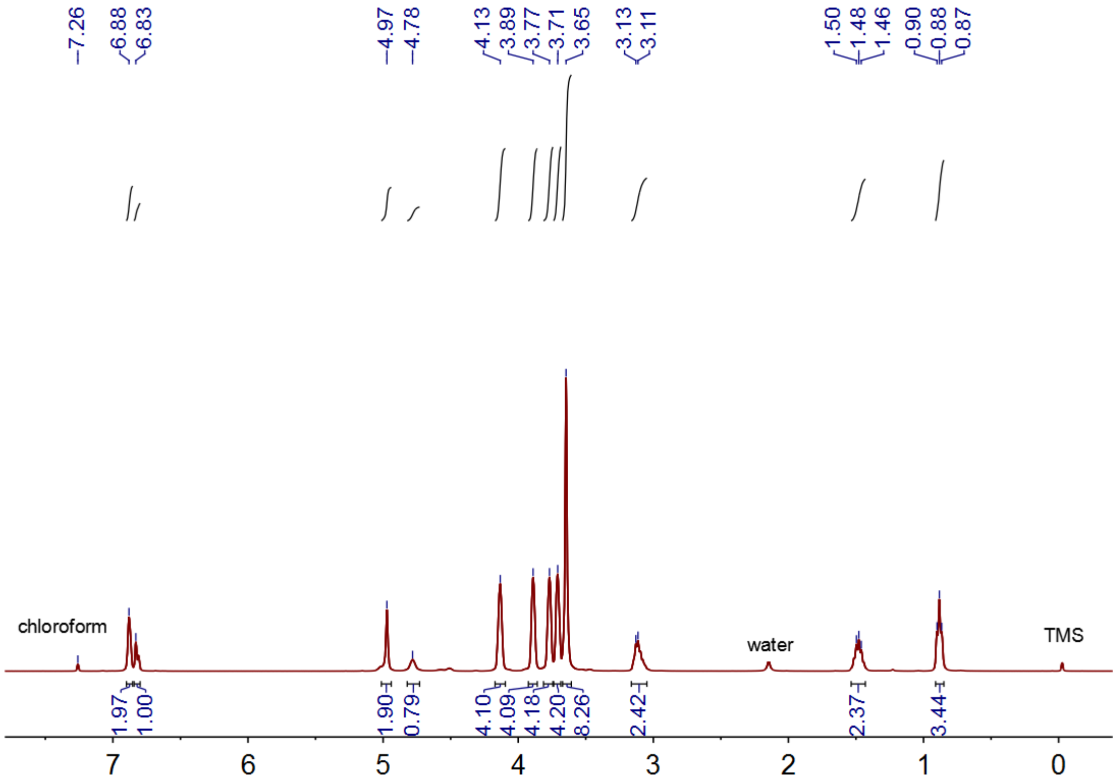

Figure S2: ^{1}H NMR spectrum (400 MHz, $CDCl_3$, room temperature) of **3a**

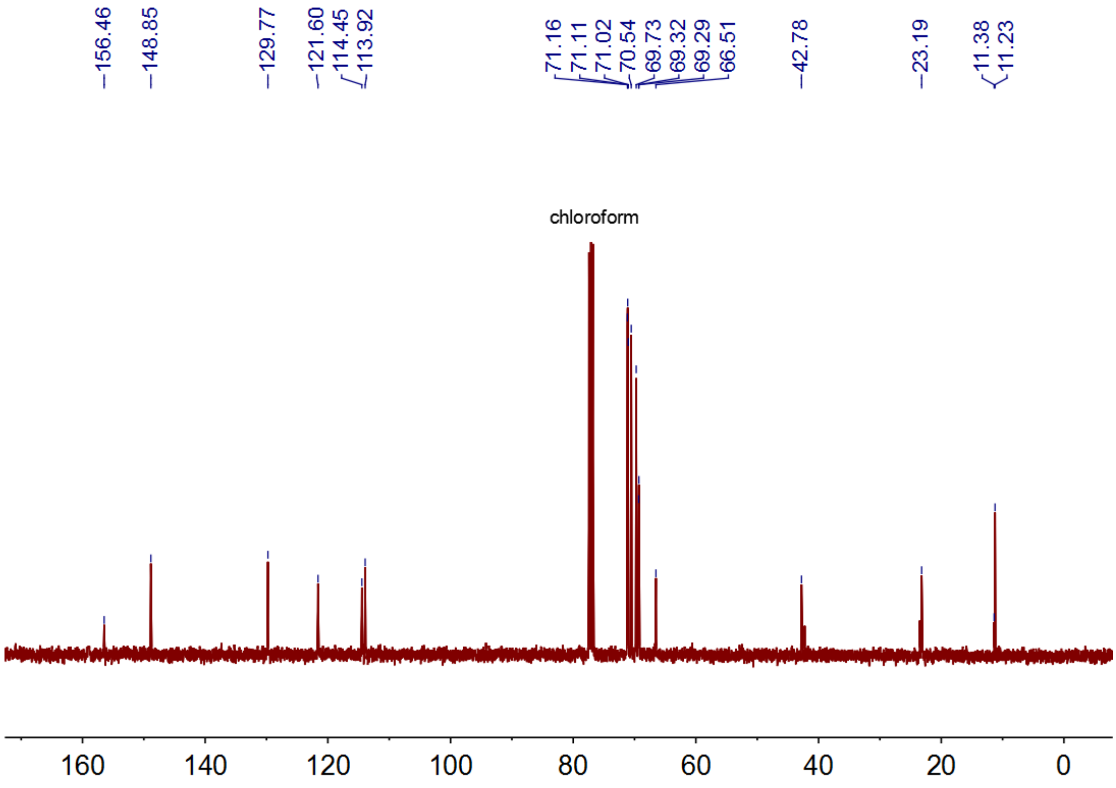

Figure S3: ^{13}C NMR spectrum (125 MHz, $CDCl_3$, room temperature) of **3a**

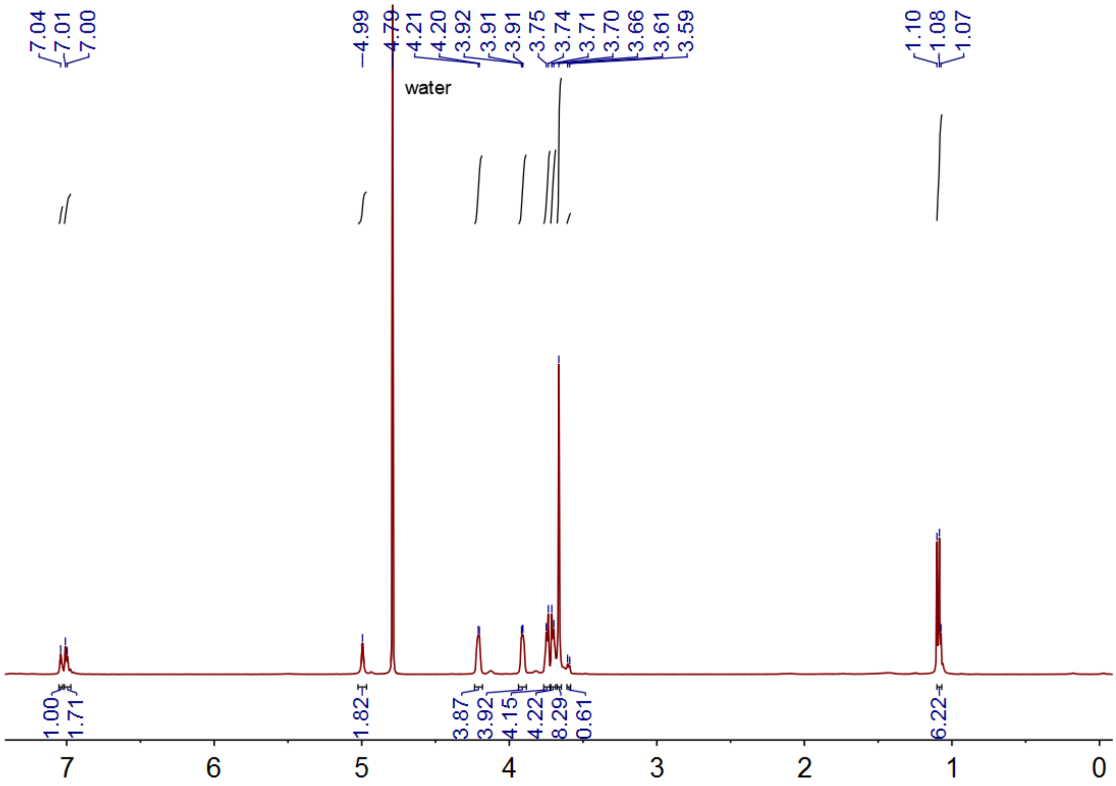

Figure S4: ^{1}H NMR spectrum (400 MHz, D_2O, room temperature) of **3b**

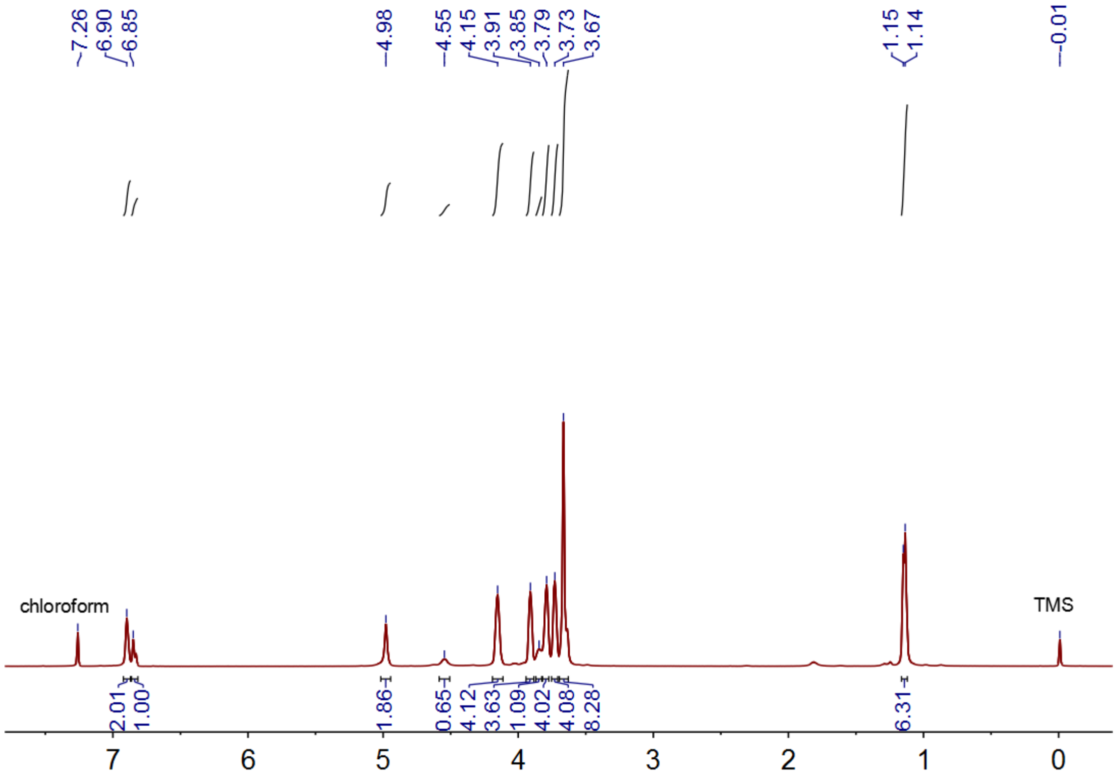

Figure S5: ^{1}H NMR spectrum (400 MHz, $CDCl_3$, room temperature) of **3b**

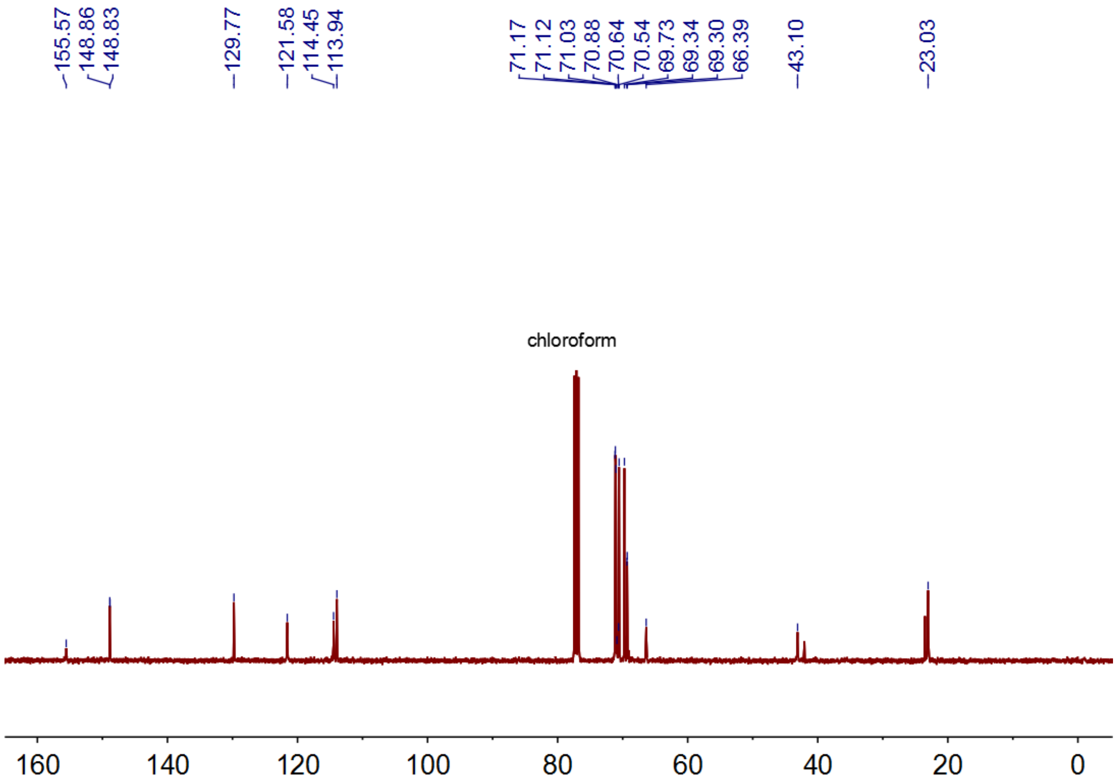

Figure S6: ^{13}C NMR spectrum (125 MHz, $CDCl_3$, room temperature) of **3b**

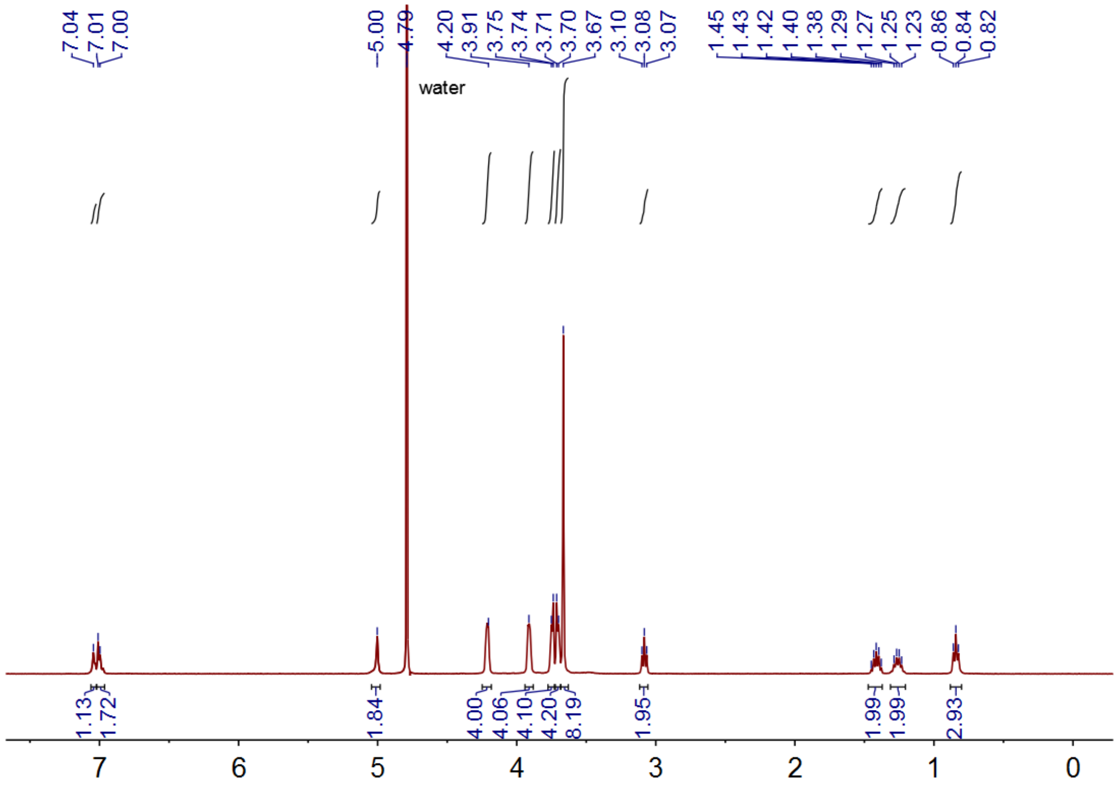

Figure S7: ^{1}H NMR spectrum (400 MHz, D_2O, room temperature) of **3c**

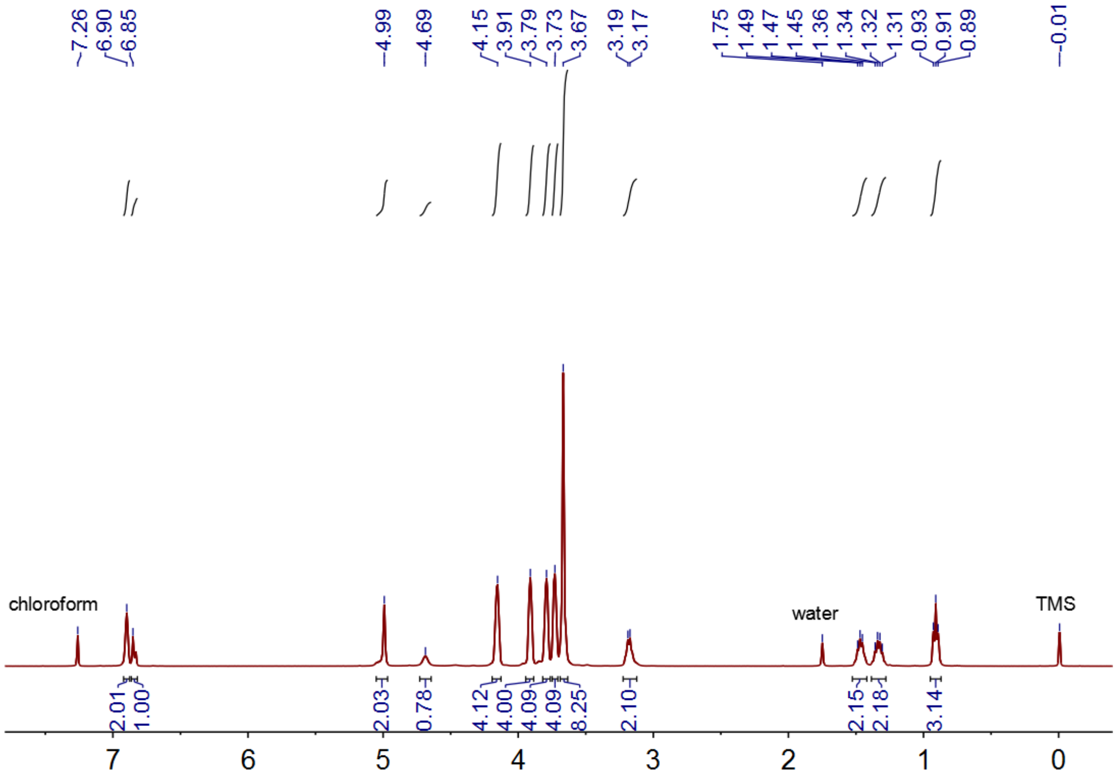

Figure S8: ^{1}H NMR spectrum (400 MHz, $CDCl_3$, room temperature) of **3c**

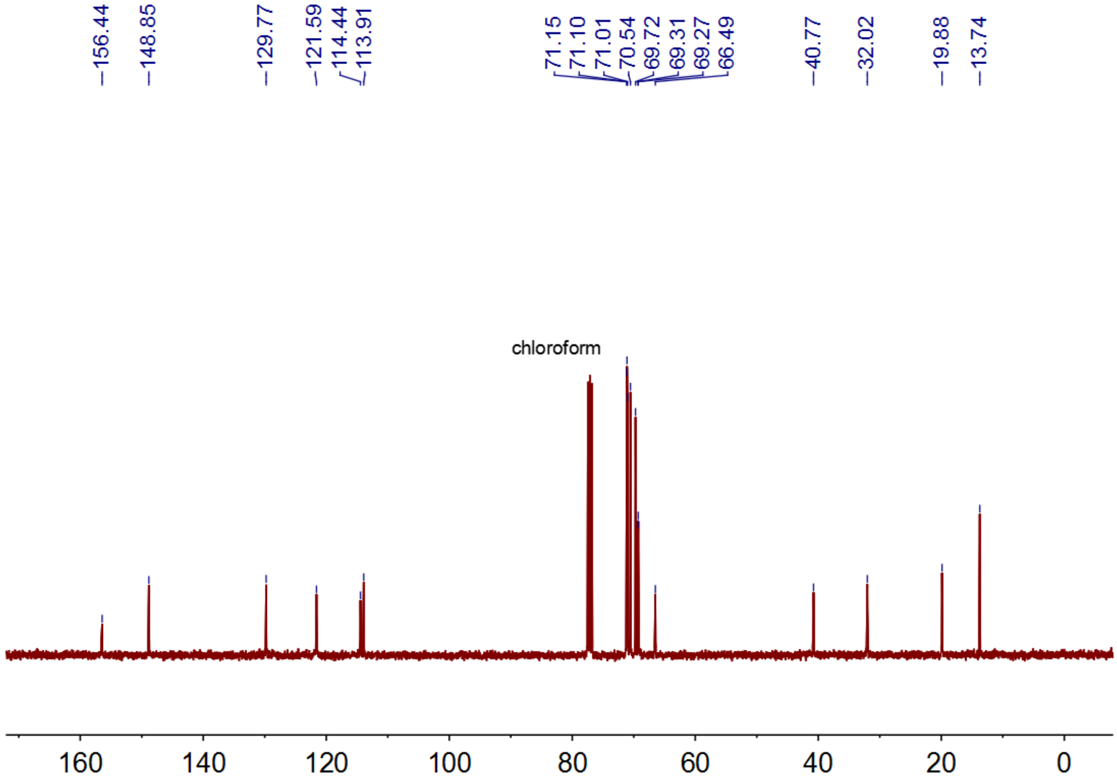

Figure S9: ^{13}C NMR spectrum (125 MHz, $CDCl_3$, room temperature) of **3c**

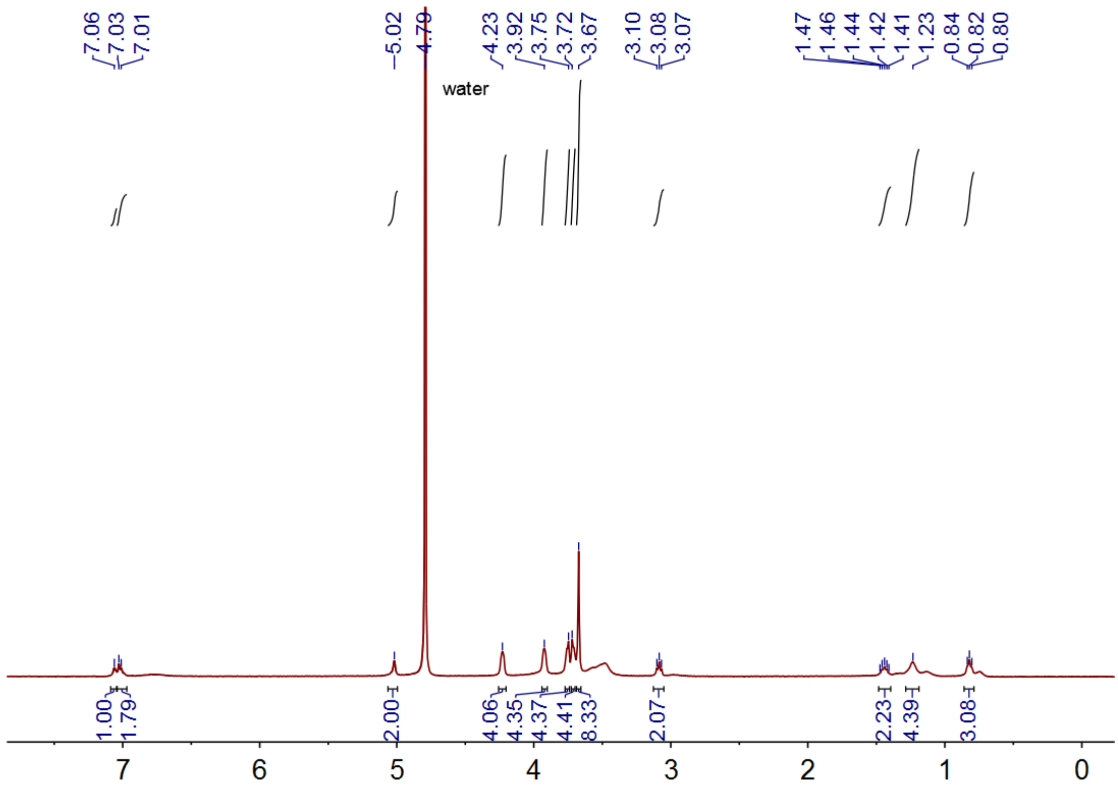

Figure S10: ^{1}H NMR spectrum (400 MHz, D_2O, room temperature) of **3d**

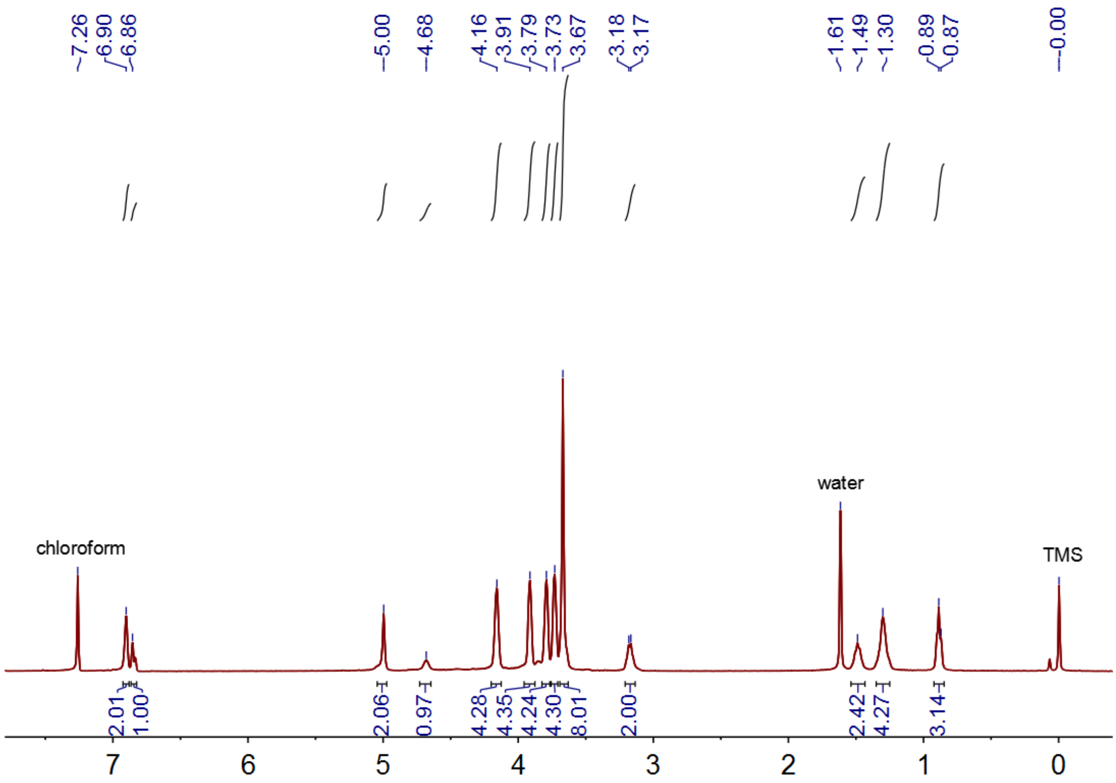

Figure S11: ^{1}H NMR spectrum (400 MHz, $CDCl_3$, room temperature) of **3d**

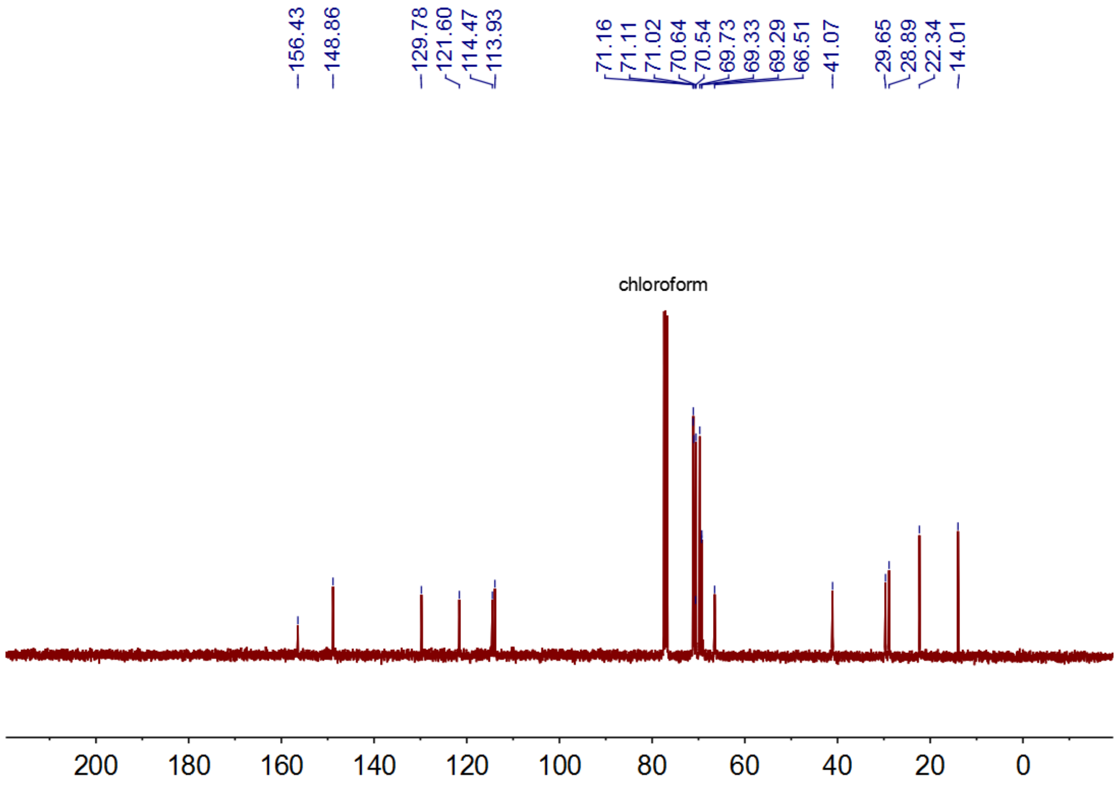

Figure S12: ^{13}C NMR spectrum (125 MHz, $CDCl_3$, room temperature) of **3d**

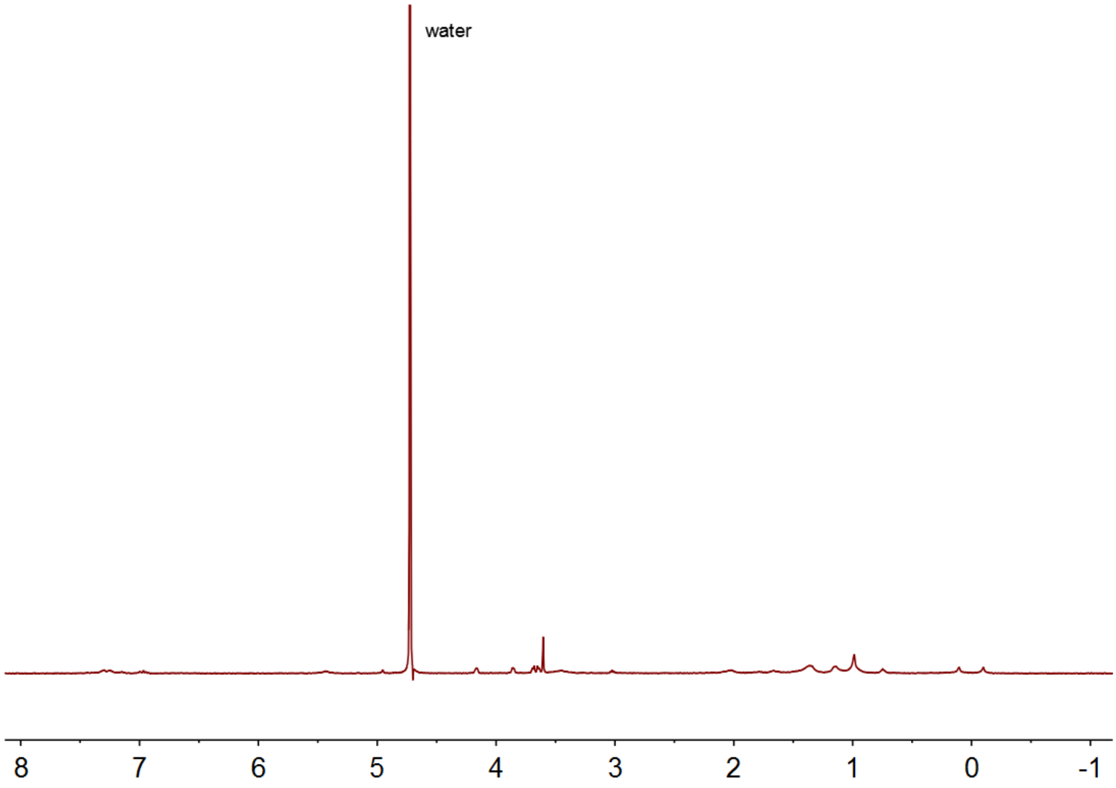

Figure S13: ^{1}H NMR spectrum (400 MHz, D_2O, room temperature) of **3e**

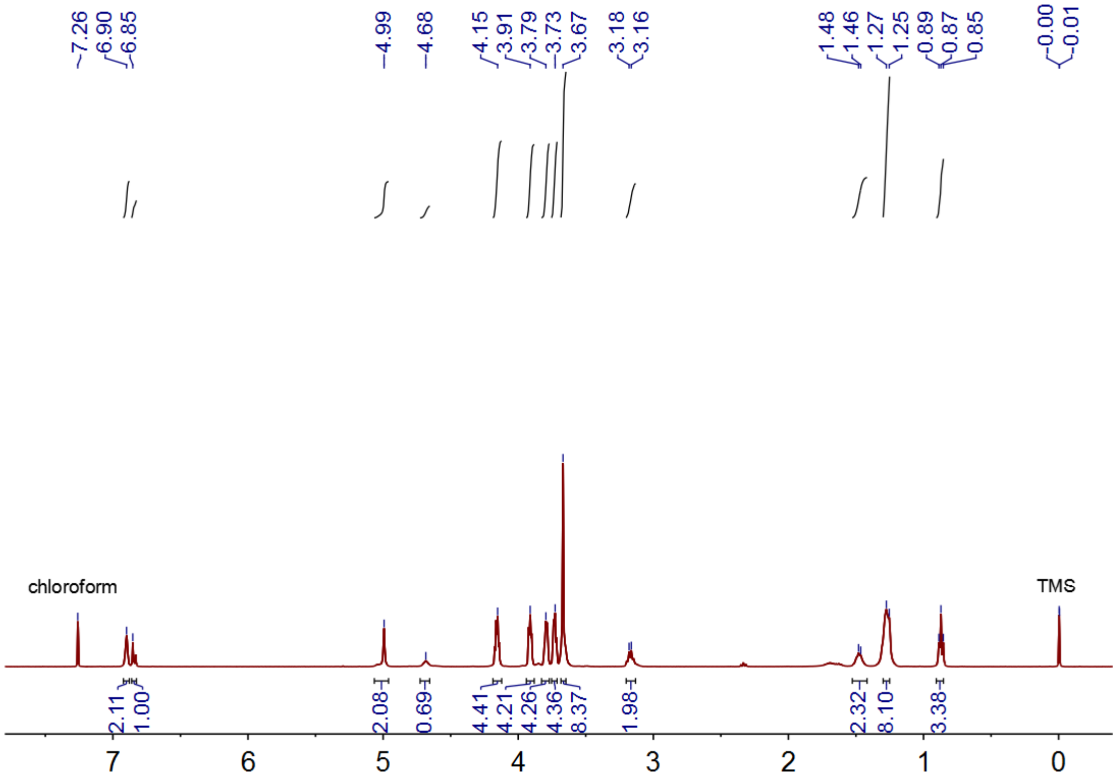

Figure S14: ^{1}H NMR spectrum (400 MHz, $CDCl_3$, room temperature) of **3e**

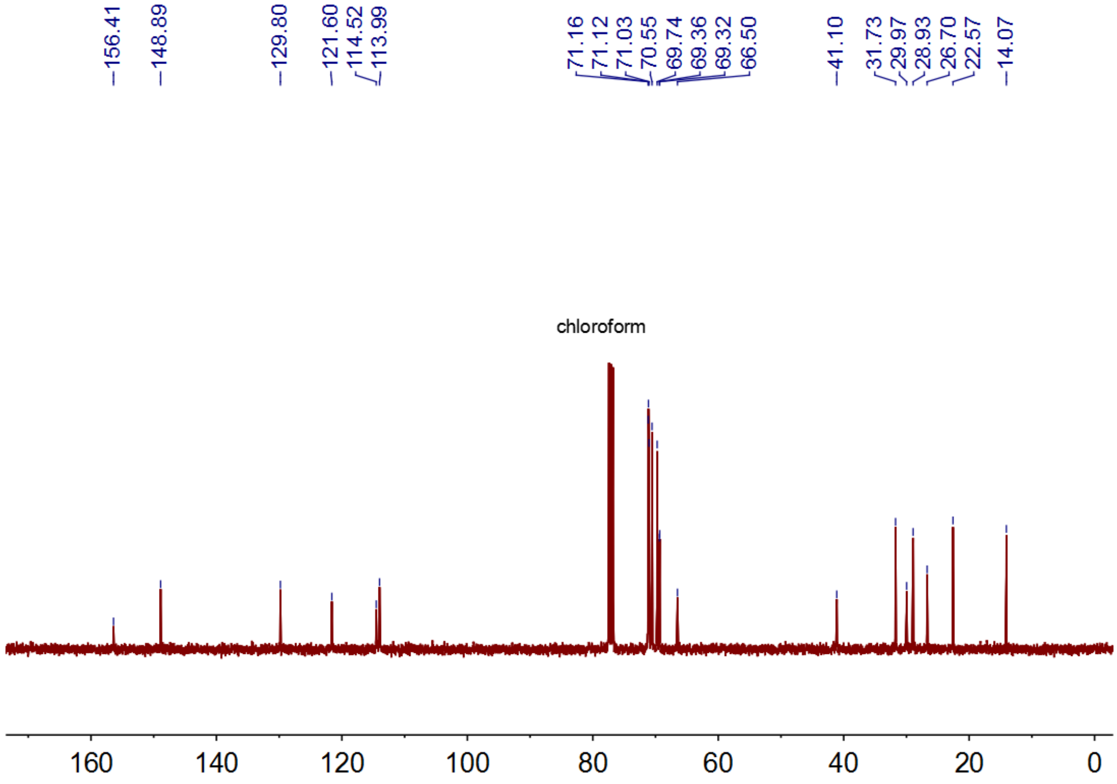

Figure S15: ^{13}C NMR spectrum (125 MHz, $CDCl_3$, room temperature) of **3e**

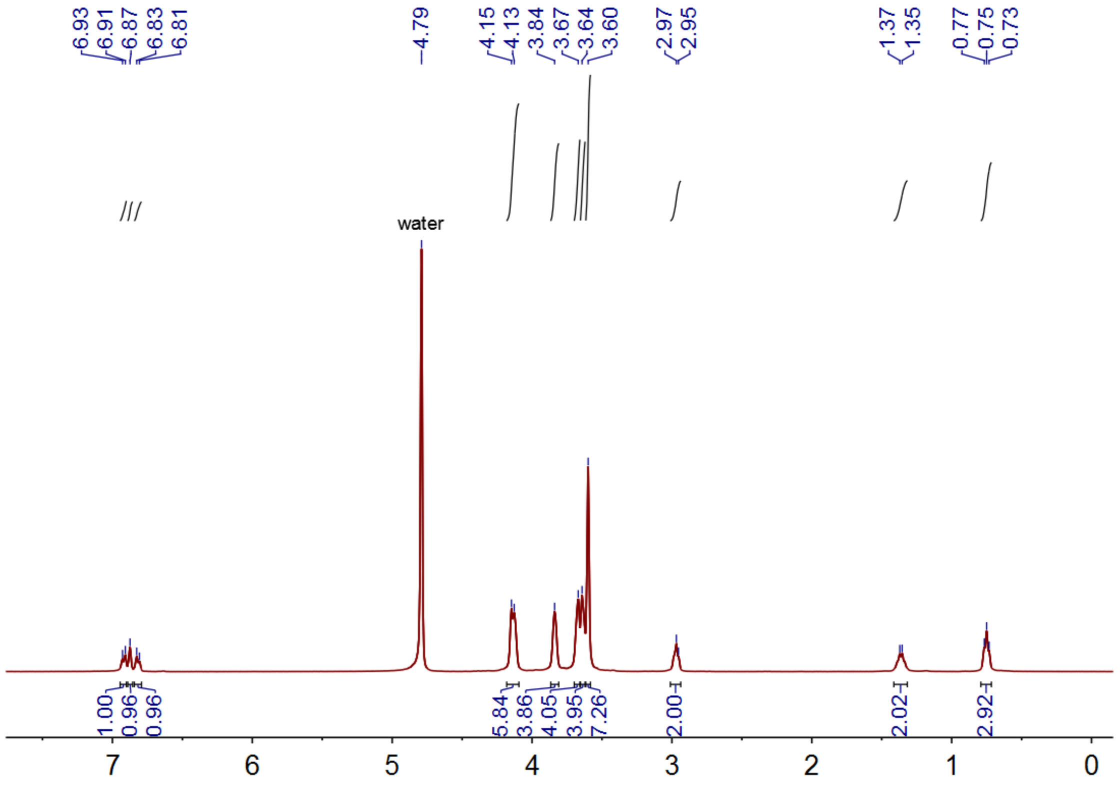

Figure S16: ^{1}H NMR spectrum (400 MHz, D_2O, room temperature) of **5a**

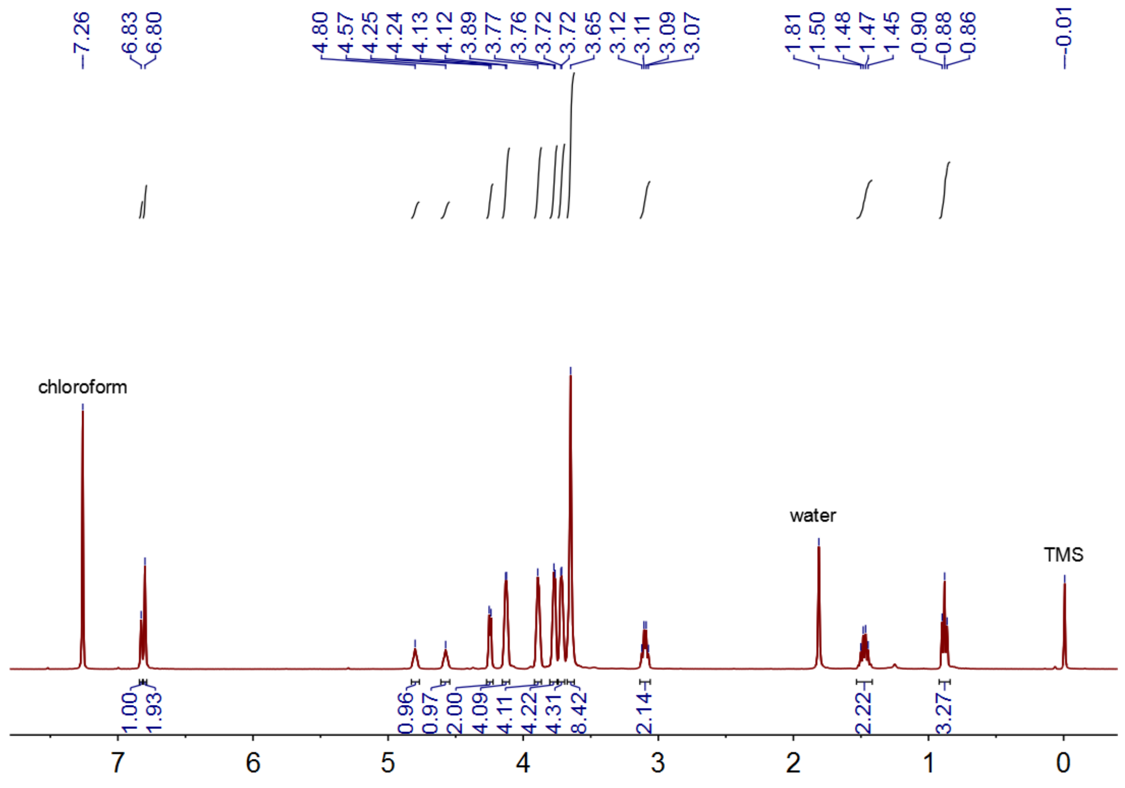

Figure S17: ^{1}H NMR spectrum (400 MHz, $CDCl_3$, room temperature) of **5a**

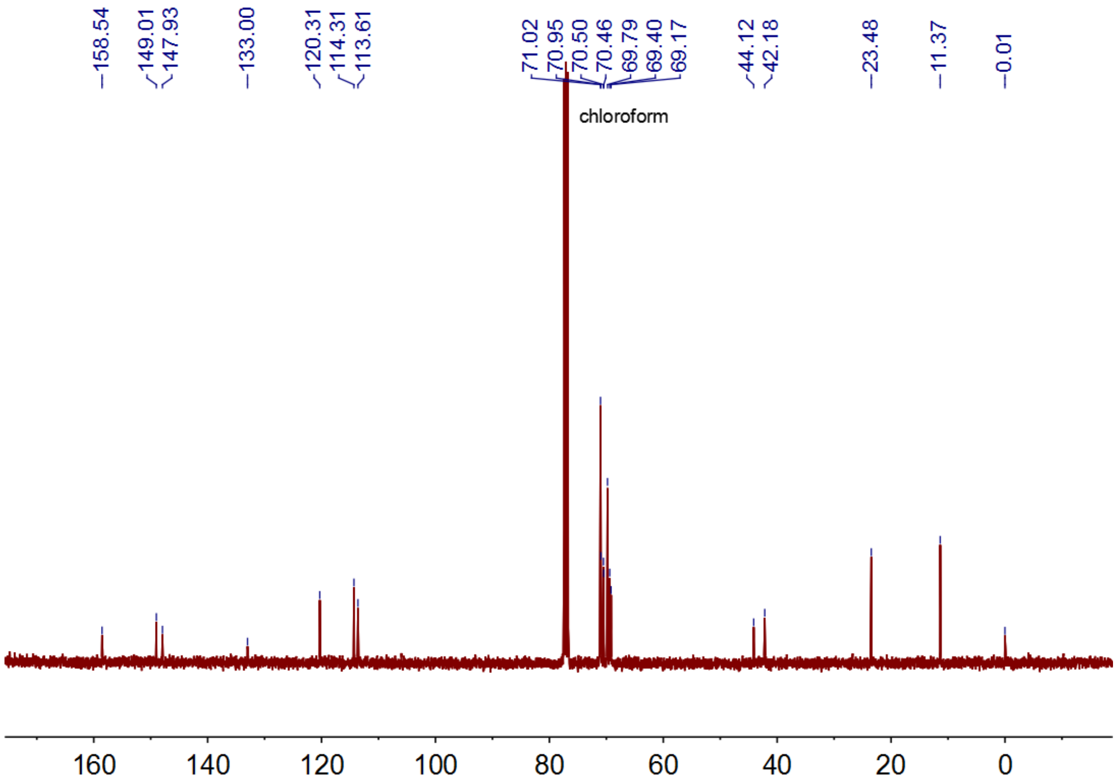

Figure S18: ^{13}C NMR spectrum (125 MHz, $CDCl_3$, room temperature) of **5a**

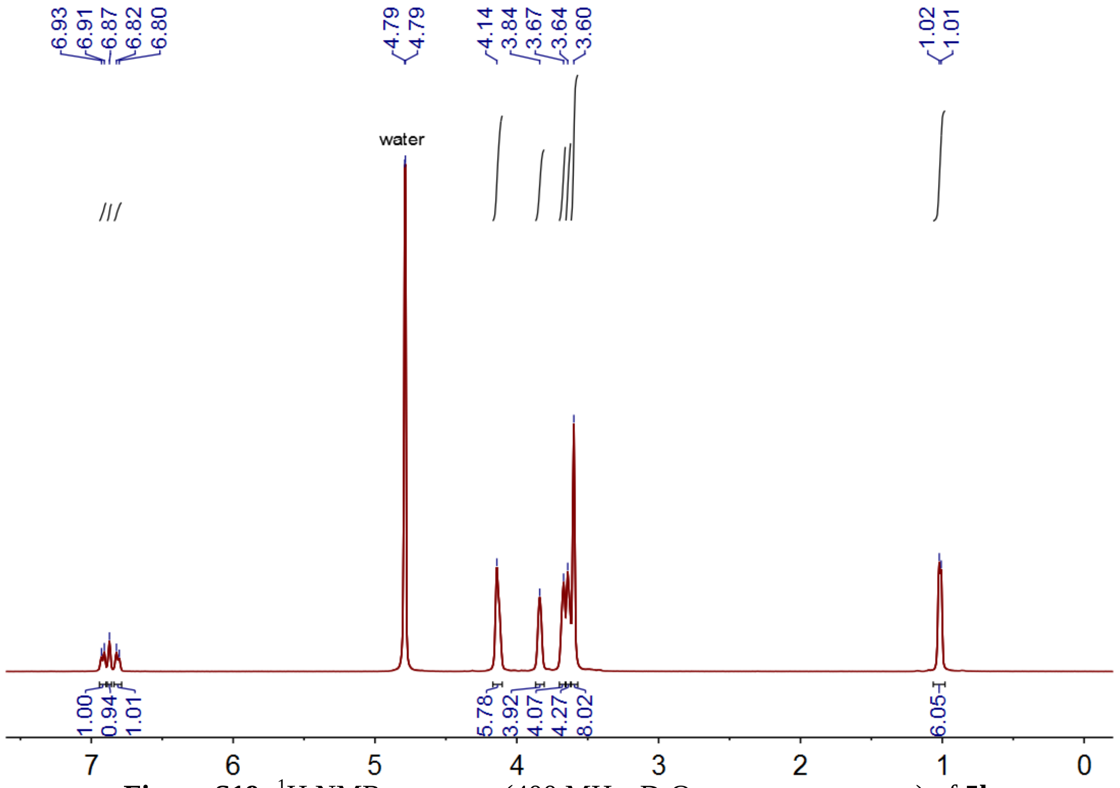

Figure S19: ^{1}H NMR spectrum (400 MHz, D_2O, room temperature) of **5b**

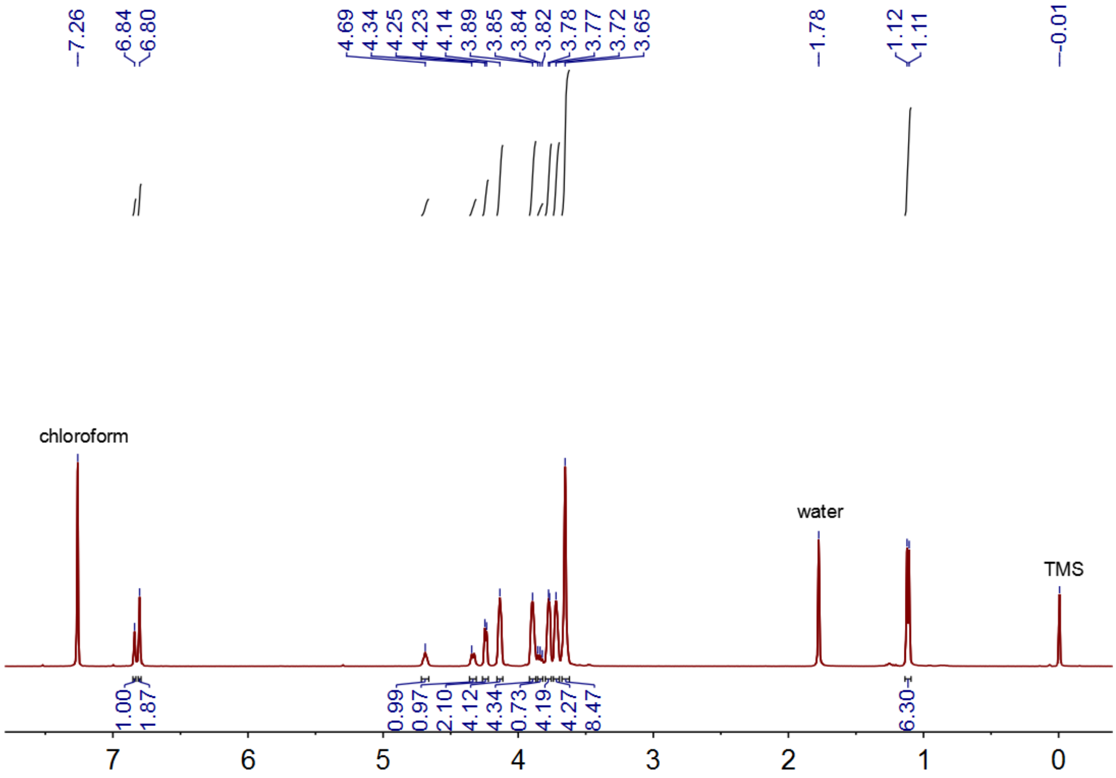

Figure S20: ^{1}H NMR spectrum (400 MHz, $CDCl_3$, room temperature) of **5b**

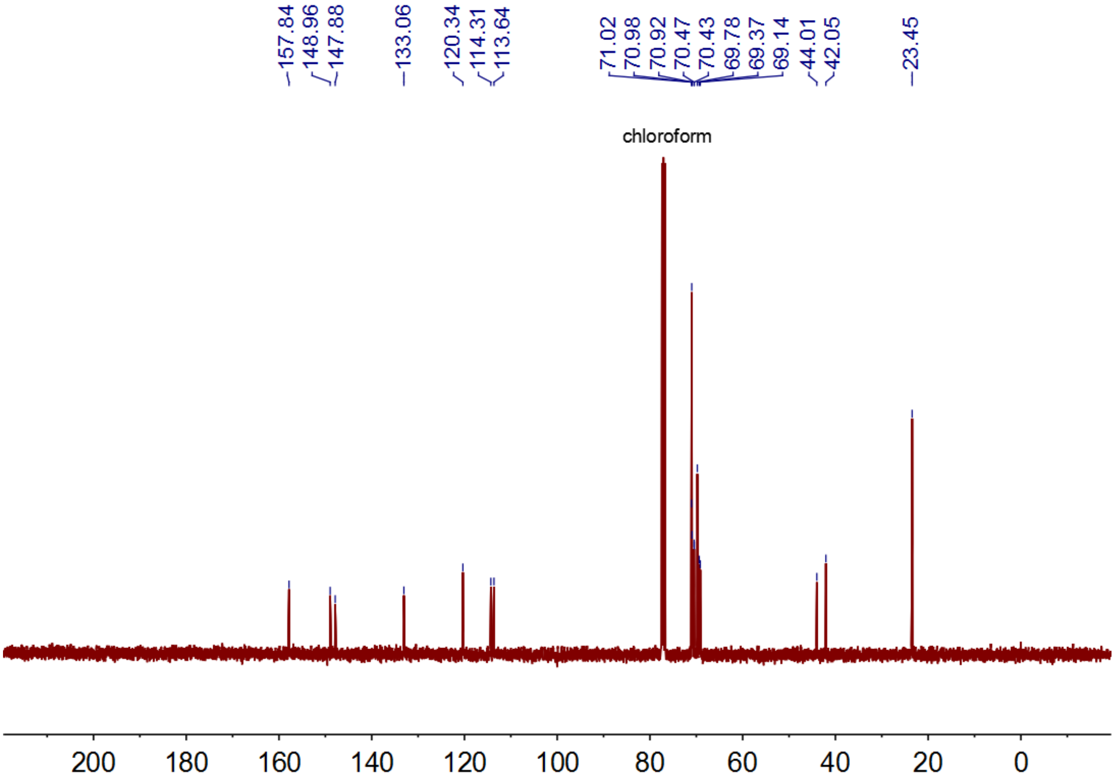

Figure S21: ^{13}C NMR spectrum (125 MHz, $CDCl_3$, room temperature) of **5b**

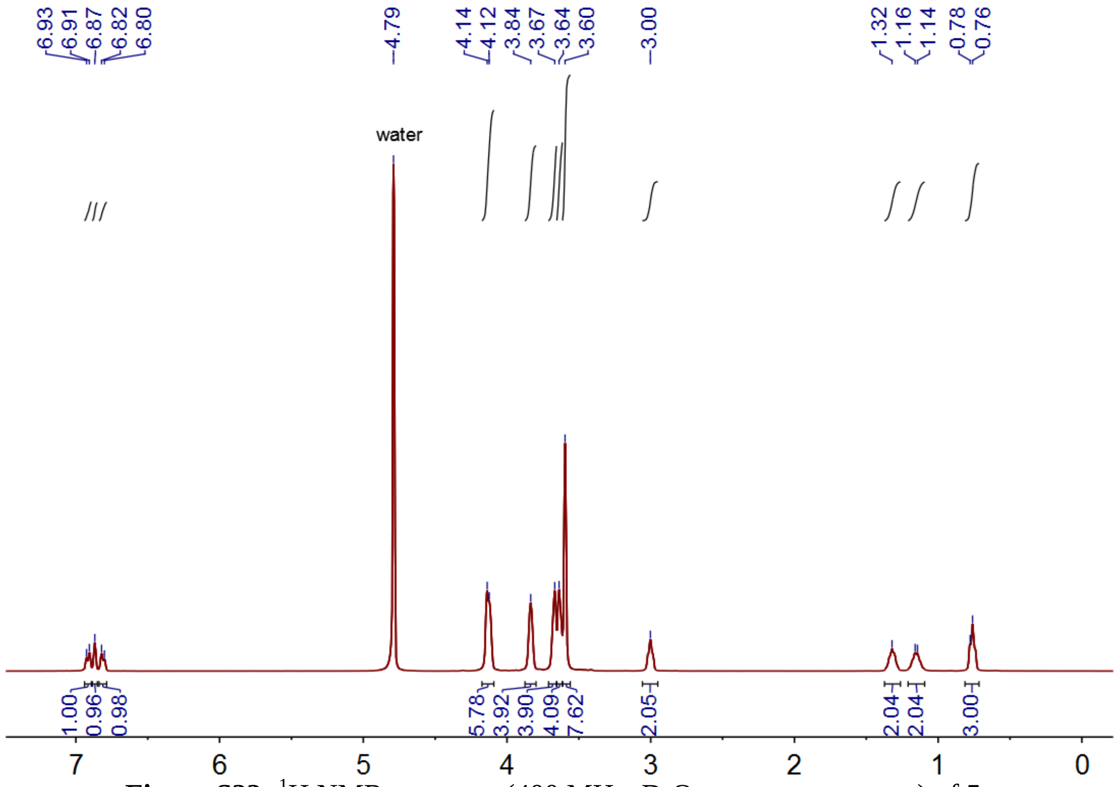

Figure S22: ^{1}H NMR spectrum (400 MHz, D_2O, room temperature) of **5c**

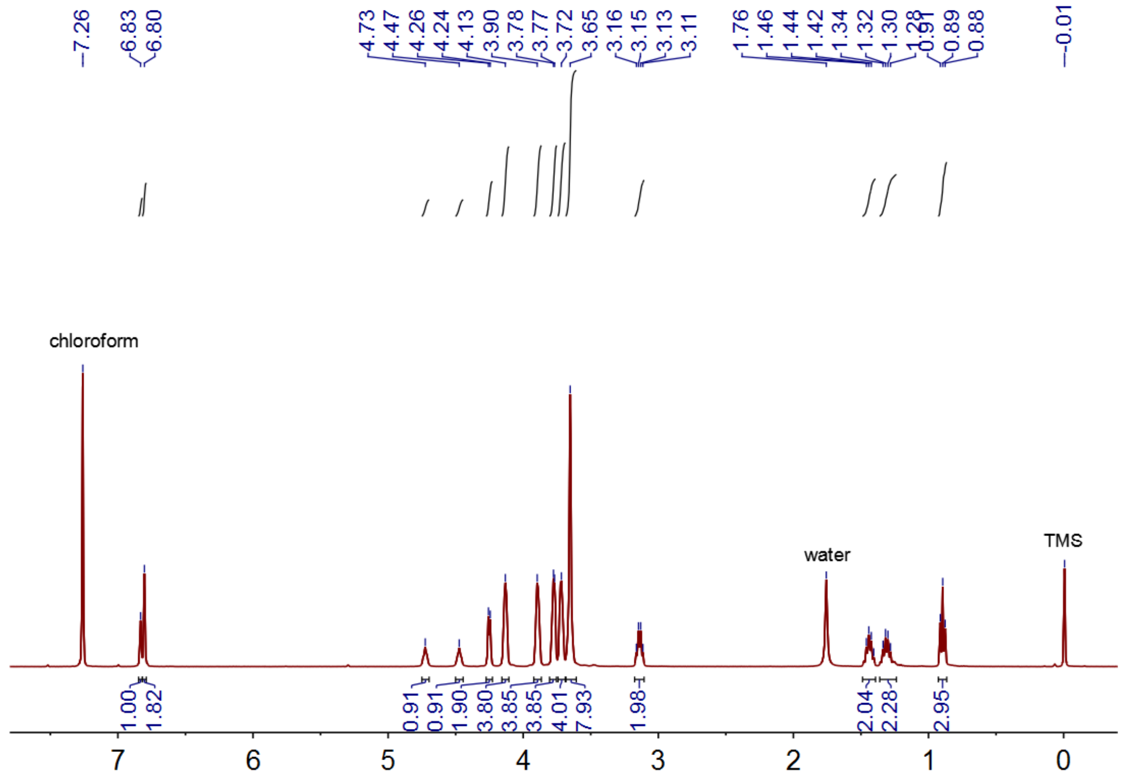

Figure S23: ^{1}H NMR spectrum (400 MHz, $CDCl_3$, room temperature) of **5c**

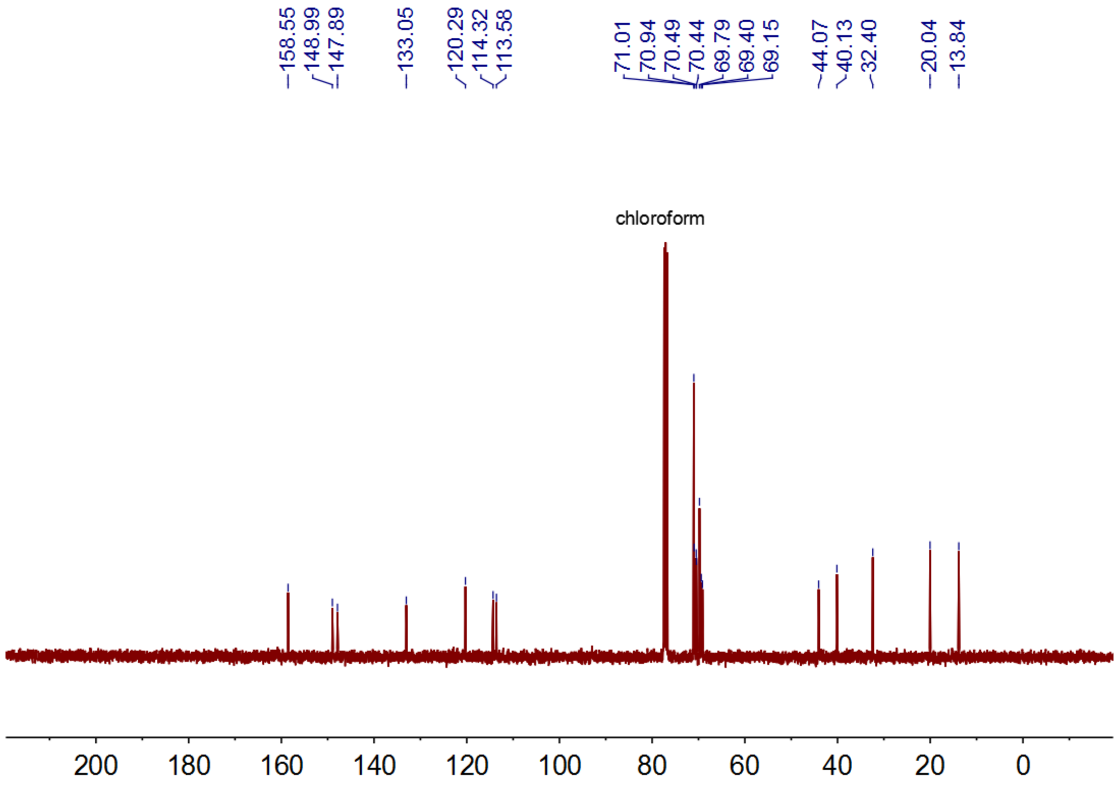

Figure S24: ^{13}C NMR spectrum (125 MHz, $CDCl_3$, room temperature) of **5c**

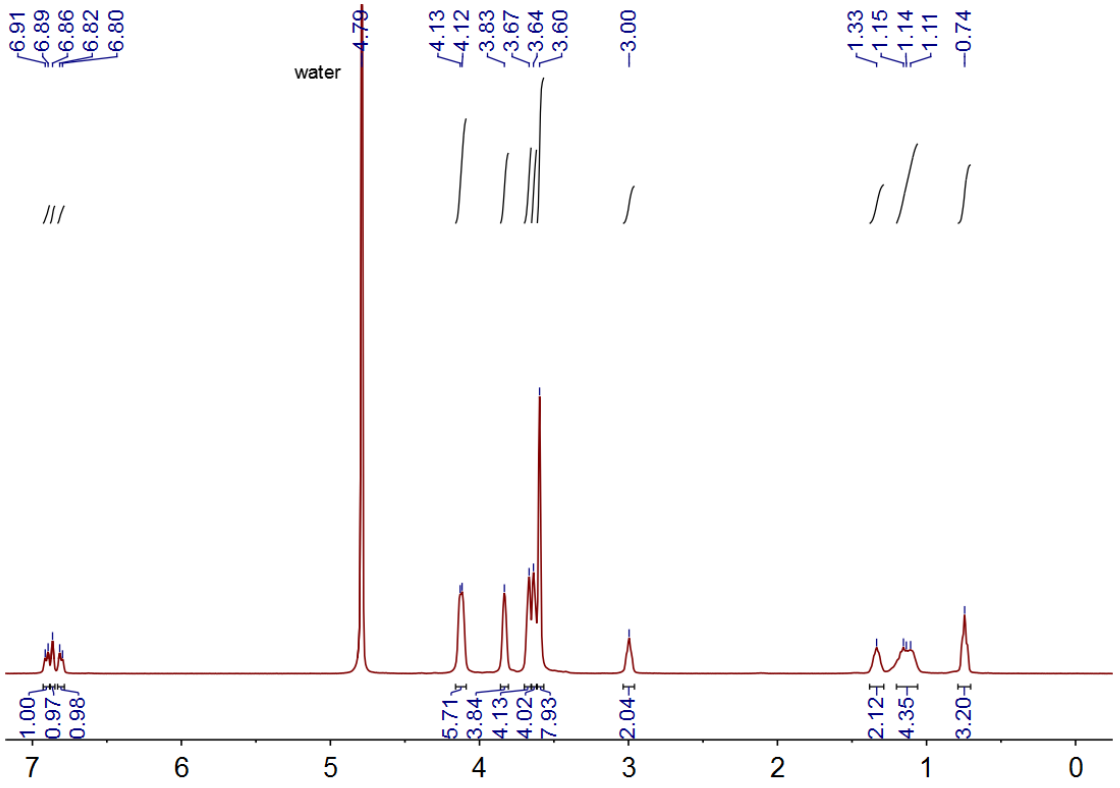

Figure S25: ^{1}H NMR spectrum (400 MHz, D_2O, room temperature) of **5d**

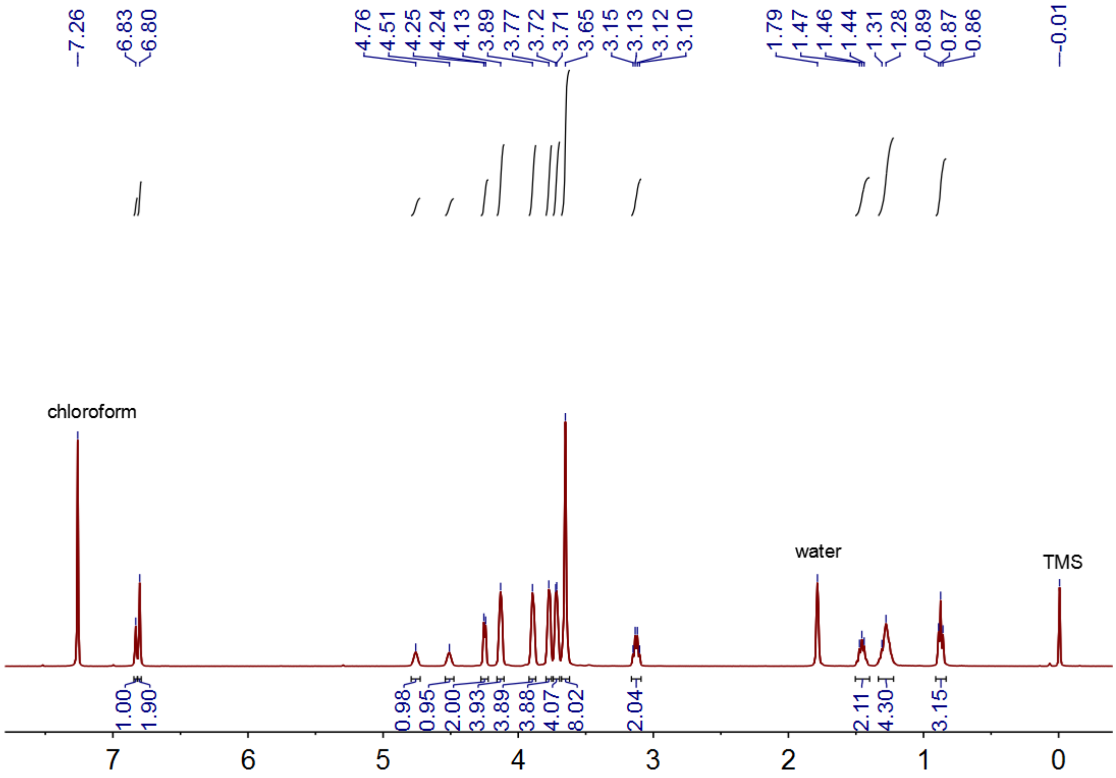

Figure S26: ^{1}H NMR spectrum (400 MHz, $CDCl_3$, room temperature) of **5d**

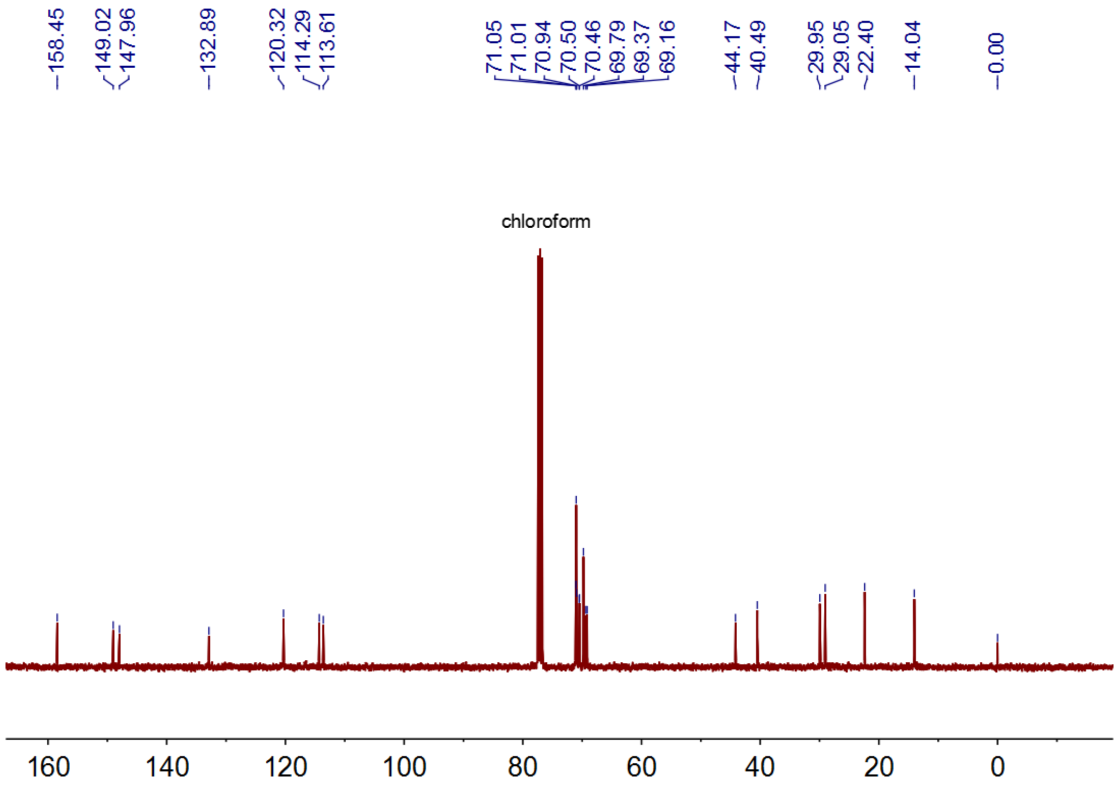

Figure S27: ^{13}C NMR spectrum (125 MHz, $CDCl_3$, room temperature) of **5d**

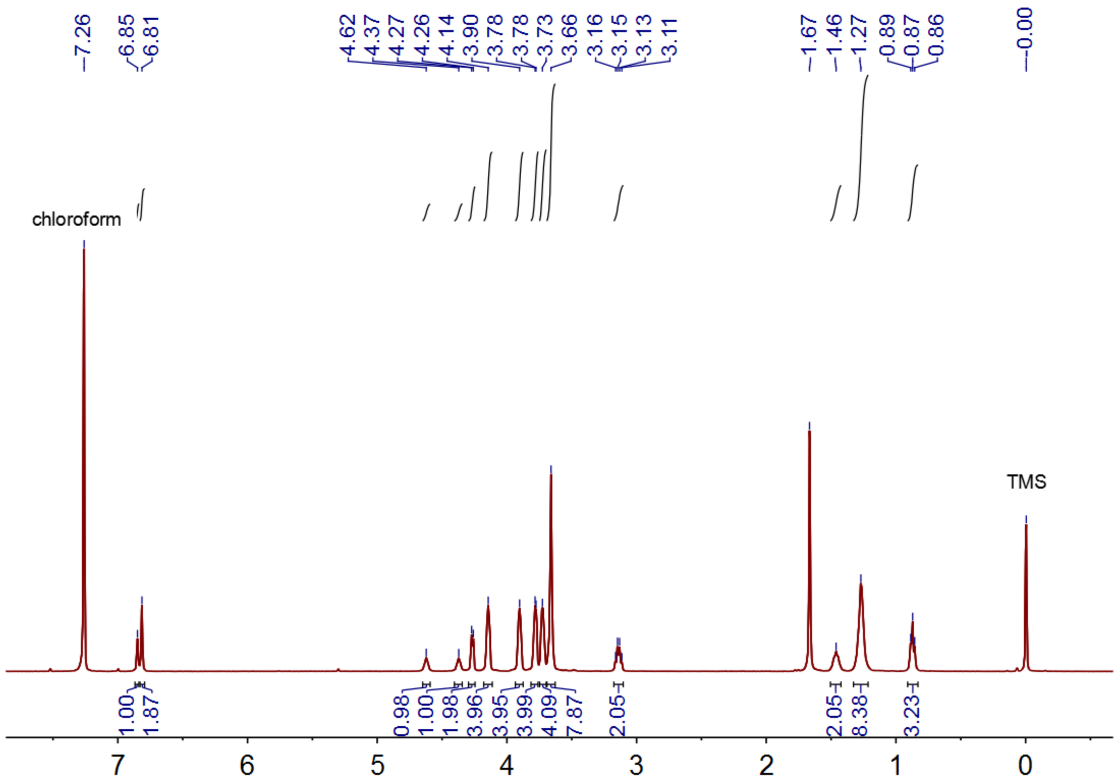

Figure S28: ^{1}H NMR spectrum (400 MHz, $CDCl_3$, room temperature) of **5e**

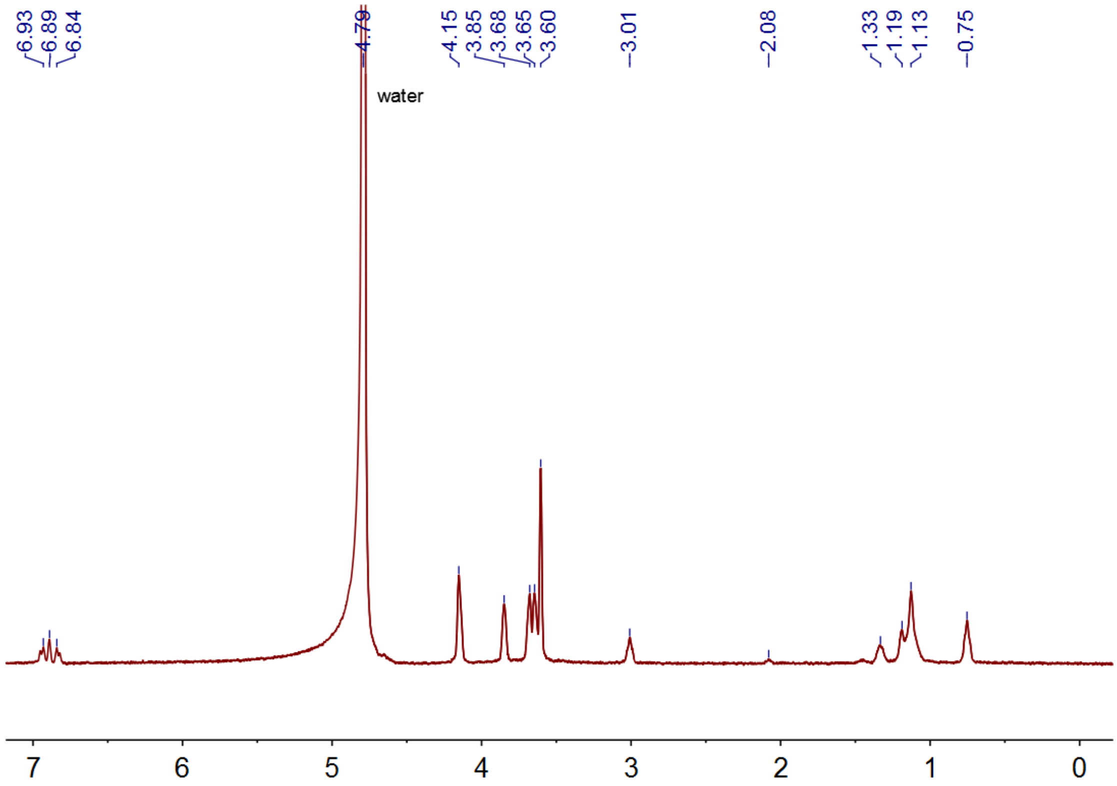

Figure S29: ^{1}H NMR spectrum (400 MHz, D_2O, room temperature) of **5e**

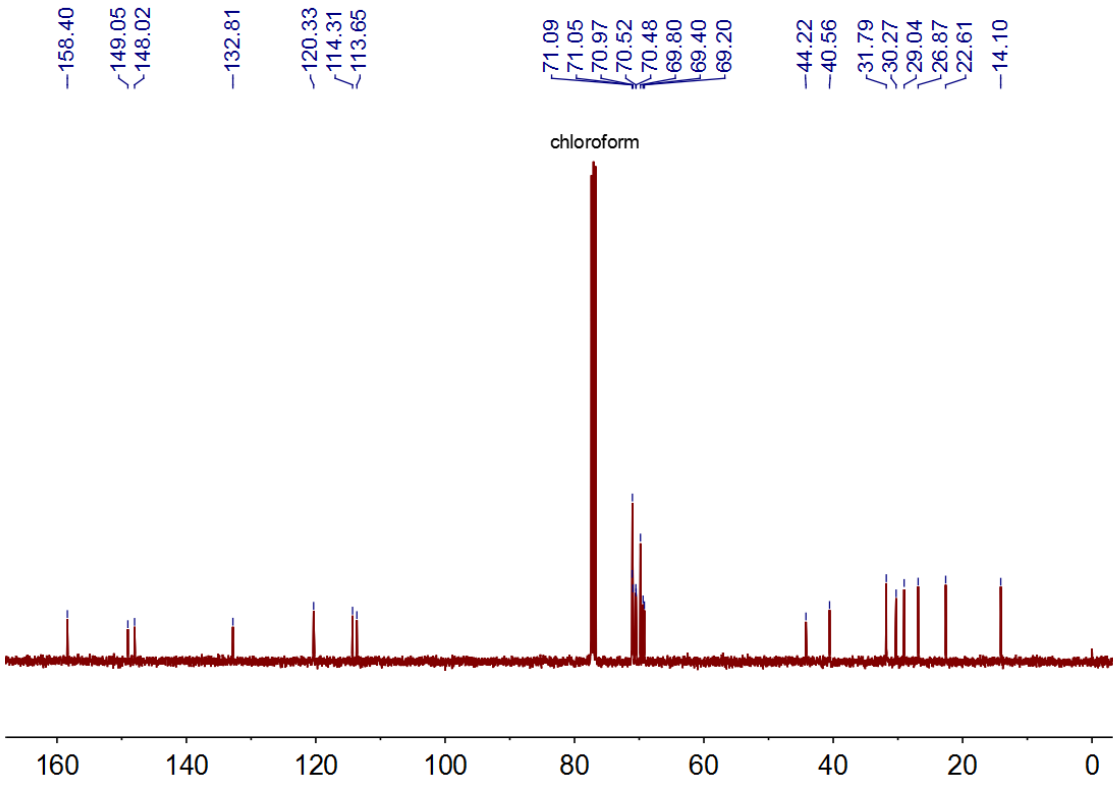

Figure S30: ^{13}C NMR spectrum (125 MHz, $CDCl_3$, room temperature) of **5e**

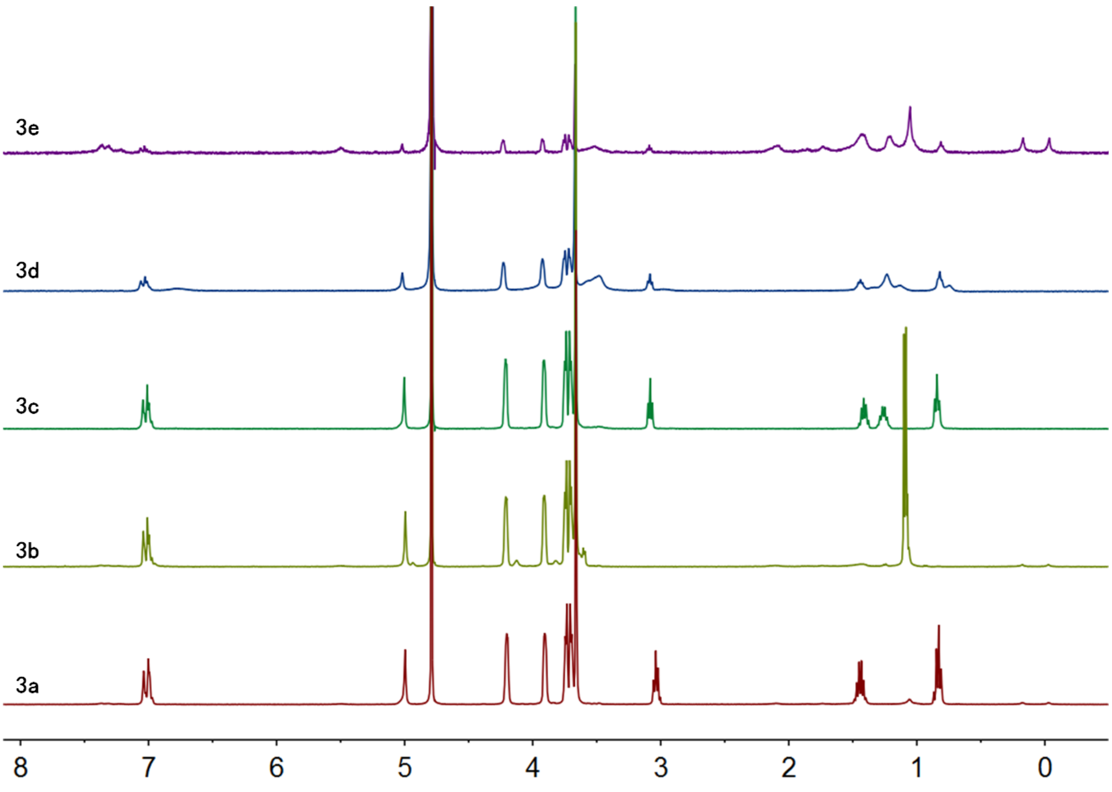

Figure S31: ^{1}H NMR spectra (400 MHz, D_2O, room temperature) of **3a-3e**

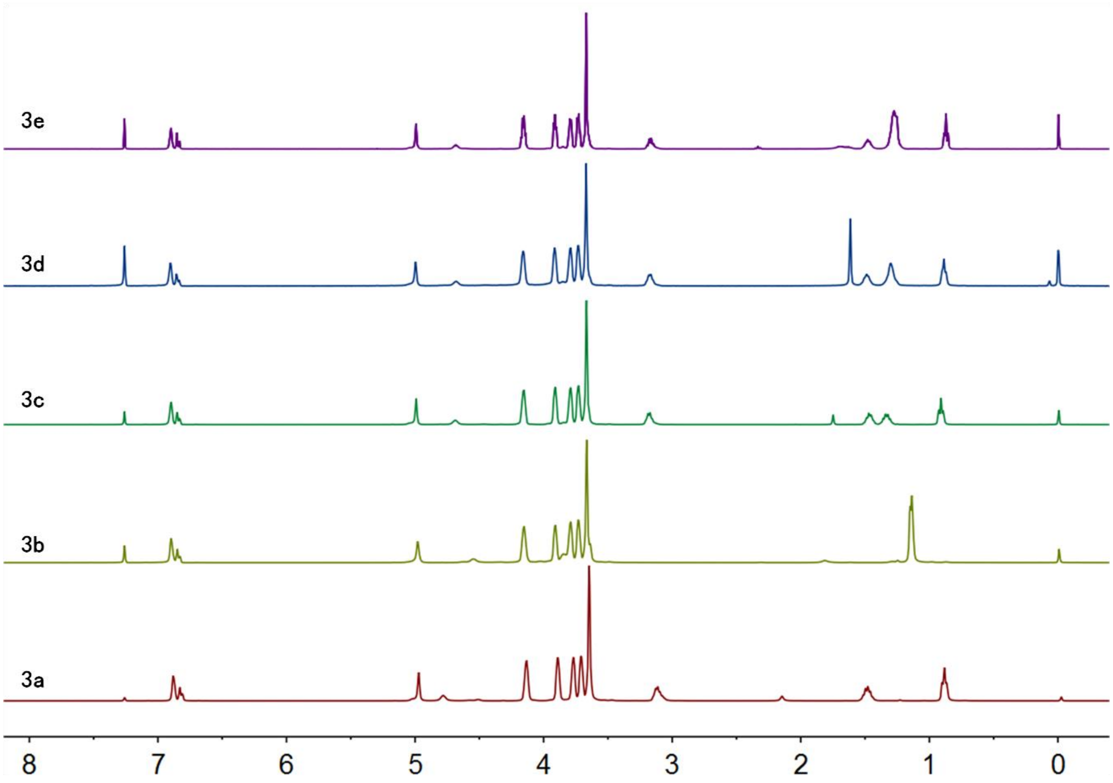

Figure S32: ^{1}H NMR spectra (400 MHz, $CDCl_3$, room temperature) of **3a-3e**

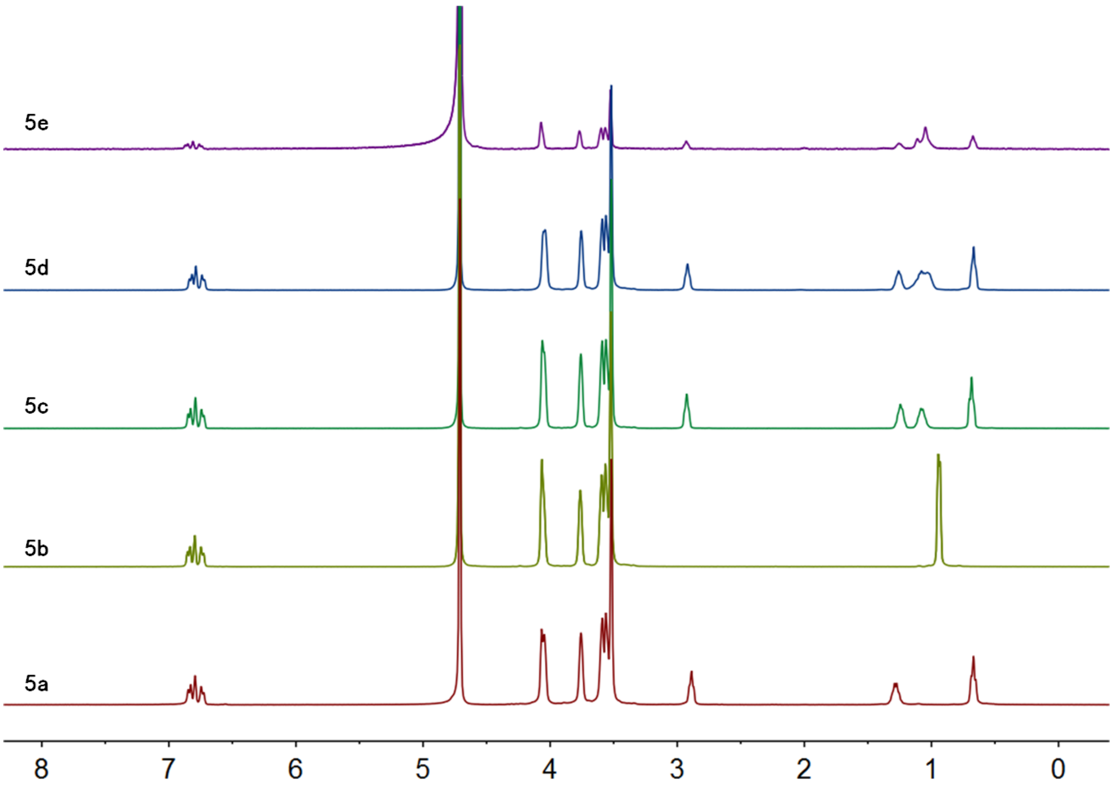

Figure S33: ^{1}H NMR spectra (400 MHz, D_2O, room temperature) of **5a-5e**

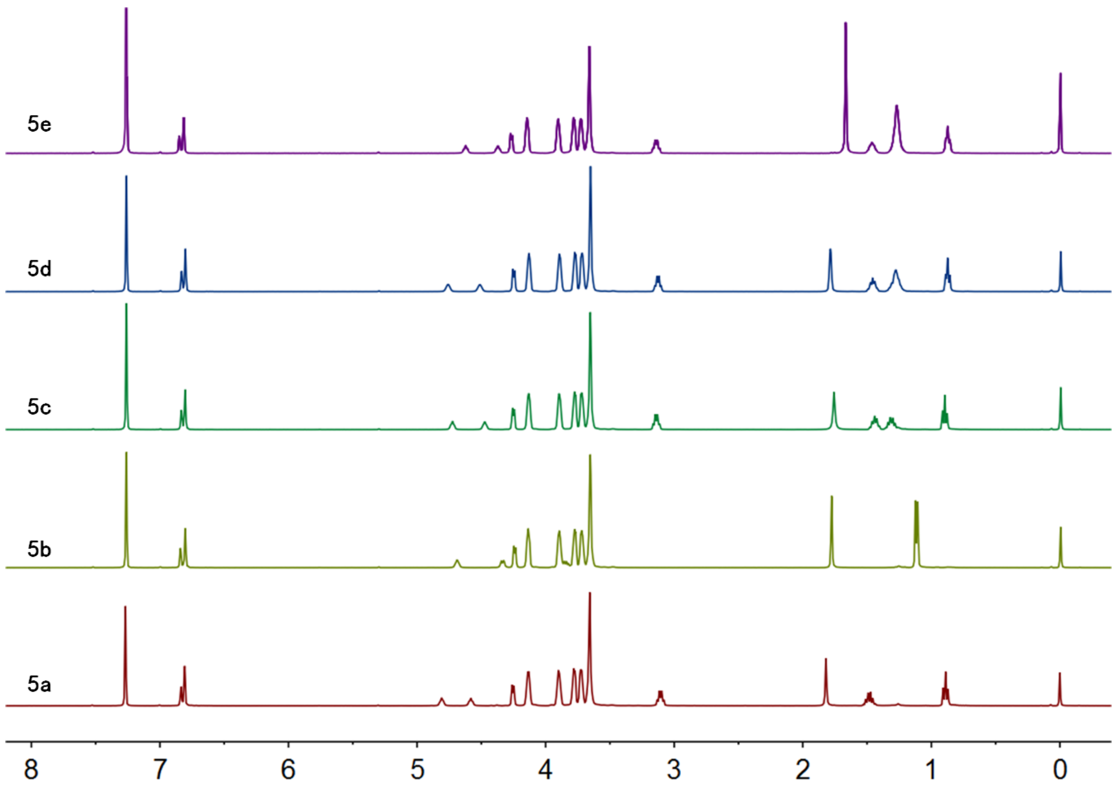

Figure S34: ^{1}H NMR spectra (400 MHz, $CDCl_3$, room temperature) of **5a-5e**

2. Solubility of 3a–e and 5a–e

Table S1: Solubility of **3a–e and 5a–e.**

Product	Solubility (mg/mL)
**3a**	26.2
**3b**	25.0
**3c**	8.0
**3d**	4.2
**3e**	0.5
**5a**	26.8
**5b**	24.0
**5c**	11.0
**5d**	7.7
**5e**	1.0

3. Variable temperature UV–vis of 5d, 3a–e.

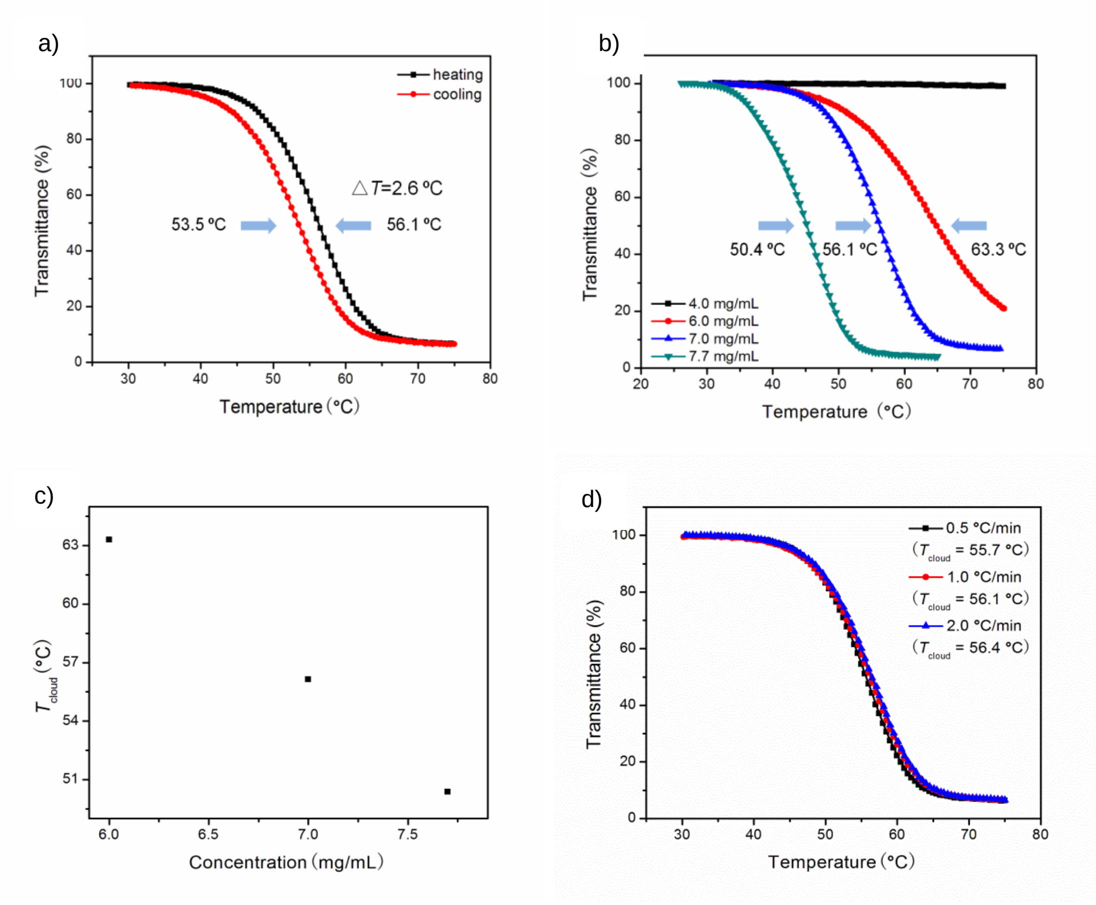

Figure S35: (a) Turbid curve for **5d** (7.0 mg/mL) measured at 550 nm. The rates for heating and cooling are 1.0 ºC/min. (b) Concentration-dependent turbidity curve for **5d** with a heating rate at 1.0 ºC/min. (c) T_{cloud} of **5d** at different concentrations with a heating rate at 1.0 ºC/min. (d) Turbidity curve for **5d** (7.0 mg/mL) measured at different heating rates.

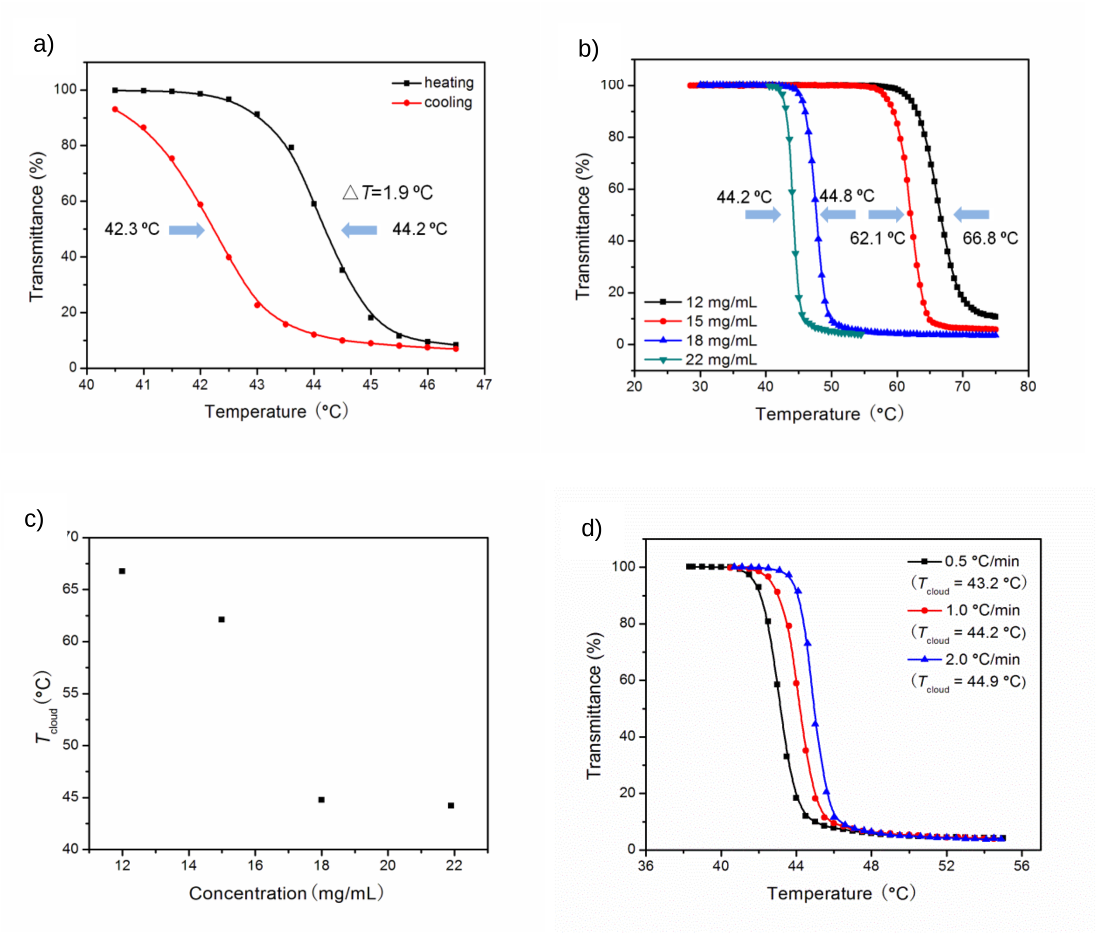

Figure S36: (a) Turbid curve for **3a** (22.0 mg/mL) measured at 550 nm. The rates for heating and cooling are 1.0 ºC/min. (b) Concentration-dependent turbidity curve for **3a** with a heating rate at 1.0 ºC/min. (c) T_{cloud} of **3a** at different concentrations with a heating rate at 1.0 ºC/min. (d) Turbidity curve for **3a** (22.0 mg/mL) measured at different heating rates.

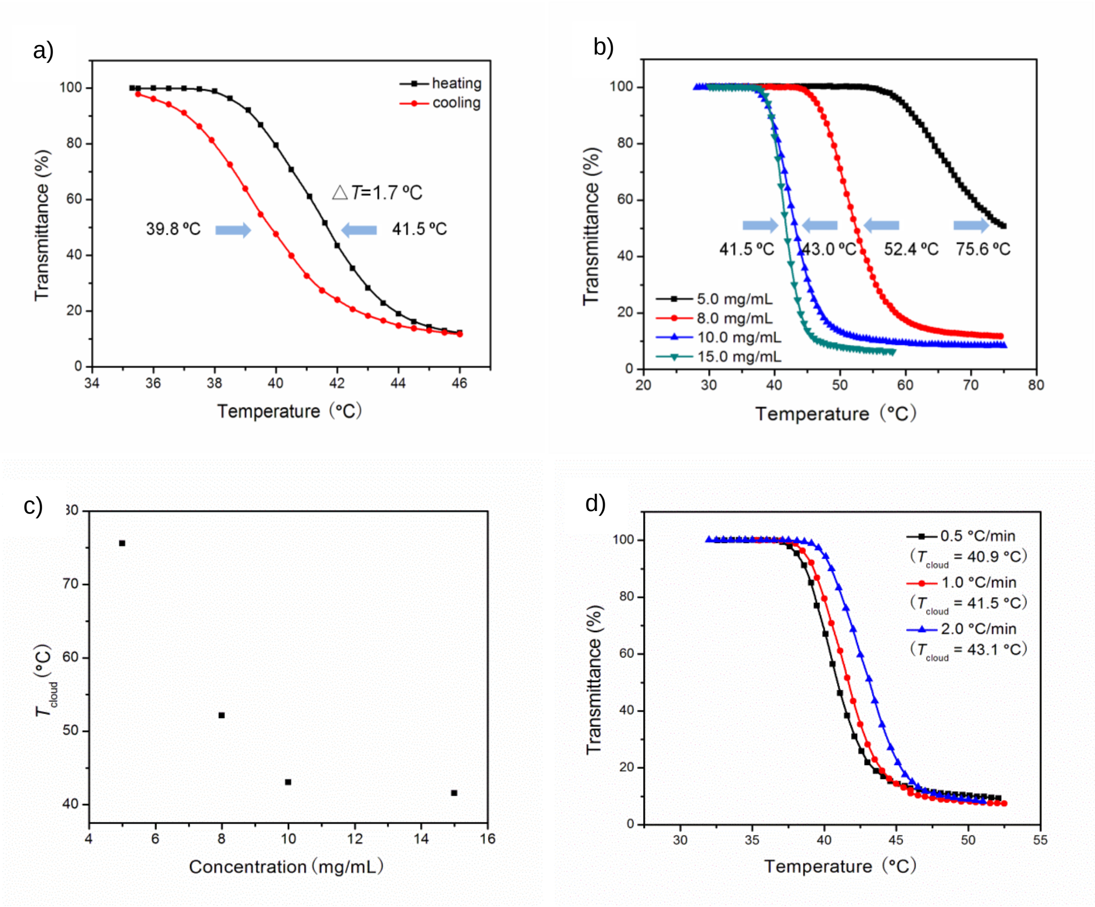

Figure S37: (a) Turbid curve for **3b** (15.0 mg/mL) measured at 550 nm. The rates for heating and cooling are 1.0 ºC/min. (b) Concentration-dependent turbidity curve for **3b** with a heating rate at 1.0 ºC/min. (c) T_{cloud} of **3b** at different concentrations with a heating rate at 1.0 ºC/min. (d) Turbidity curve for **3b** (15.0 mg/mL) measured at different heating rates.

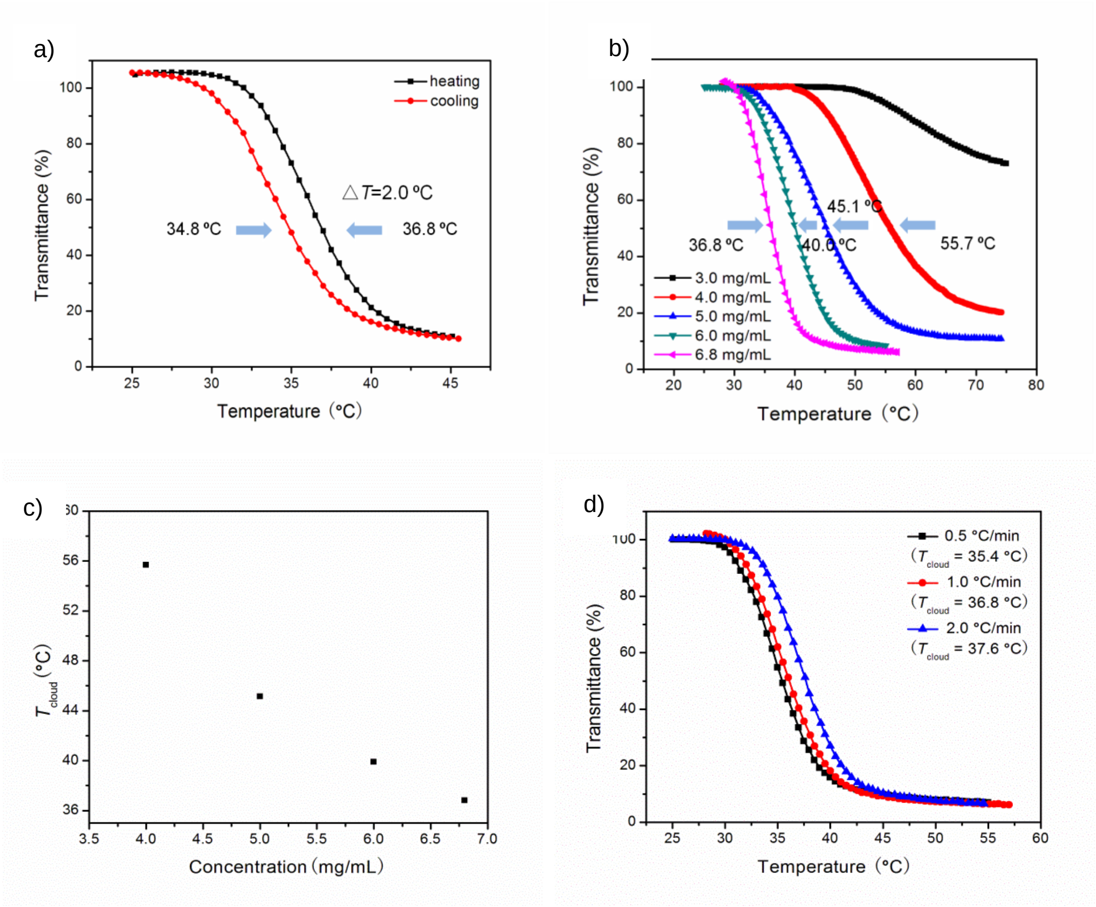

Figure S38: (a) Turbid curve for **3c** (6.8 mg/mL) measured at 550 nm. The rates for heating and cooling are 1.0 ºC/min. (b) Concentration-dependent turbidity curve for **3c** with a heating rate at 1.0 ºC/min. (c) T_{cloud} of **3c** at different concentrations with a heating rate at 1.0 ºC/min. (d) Turbidity curve for **3c** (6.8 mg/mL) measured at different heating rates.

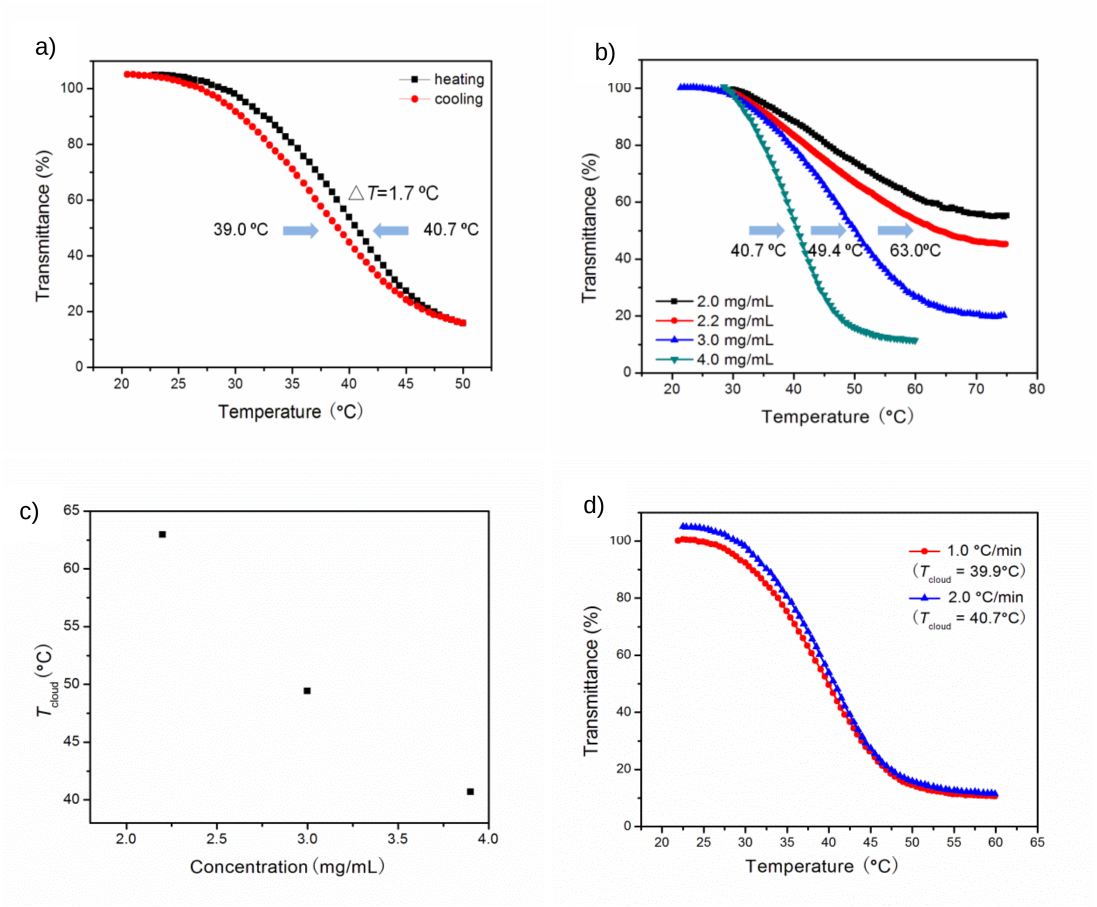

Figure S39: (a) Turbid curve for **3d** (4.0 mg/mL) measured at 550 nm. The rates for heating and cooling are 1.0 ºC/min. (b) Concentration-dependent turbidity curve for **3d** with a heating rate at 1.0 ºC/min. (c) T_{cloud} of **3d** at different concentrations with a heating rate at 1.0 ºC/min. (d) Turbidity curve for **3d** (4.0 mg/mL) measured at different heating rates.

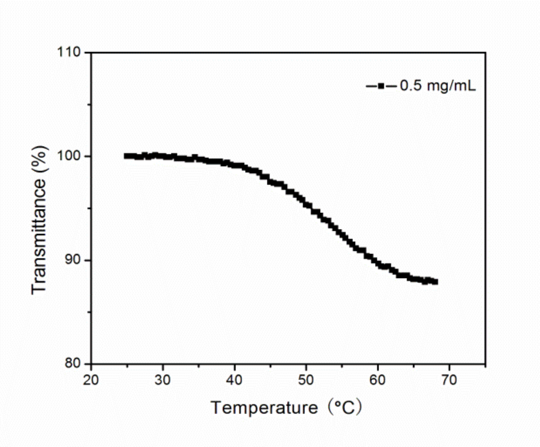

Figure S40: Variable temperature UV curve of **3e**

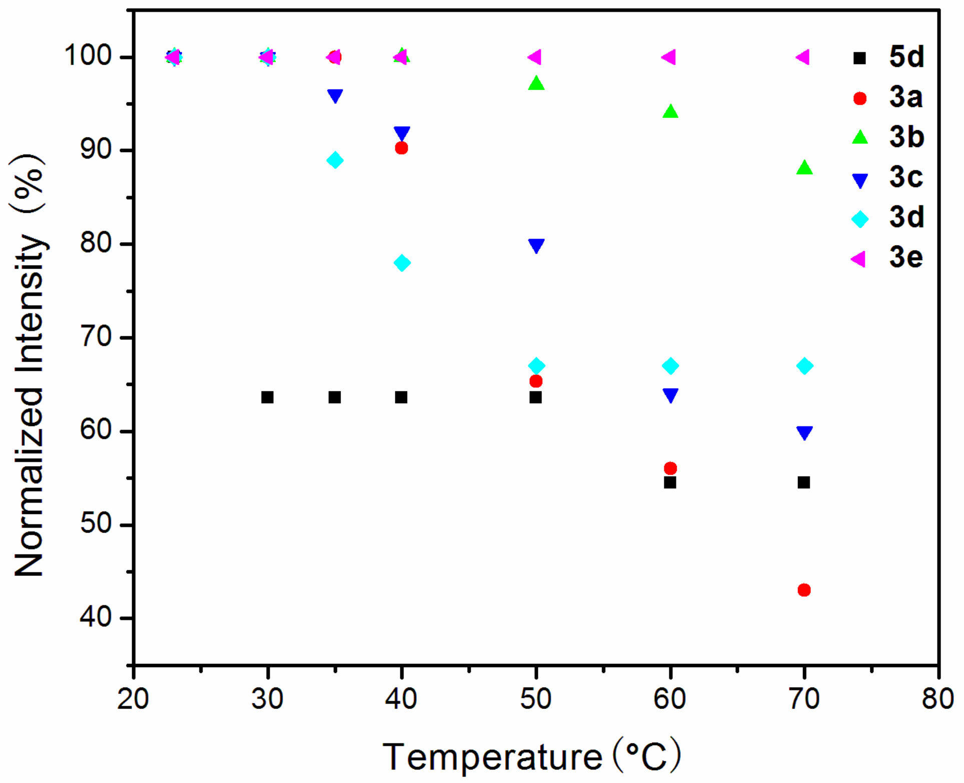

Figure S41: Temperature-dependent normalized intensity for **5d, 3a–d**.

4. Variable temperature ^{1}H NMR of 5d, 3a–e.

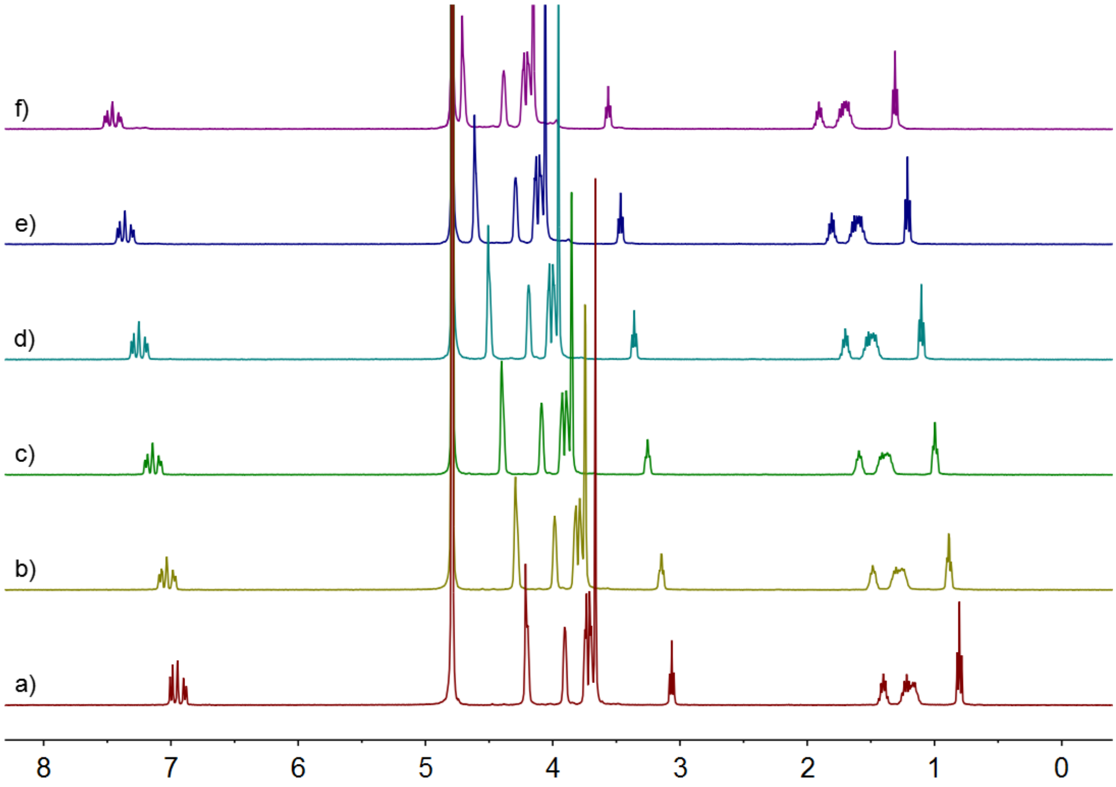

Figure S42: Temperature-dependent ^{1}H NMR spectra (400 MHz, D_2O) of **5d** (the concentration is 6 mg/mL). a) 25 ºC; b) 30 ºC; c) 40 ºC; d) 50 ºC; e) 60 ºC; f) 70 ºC.

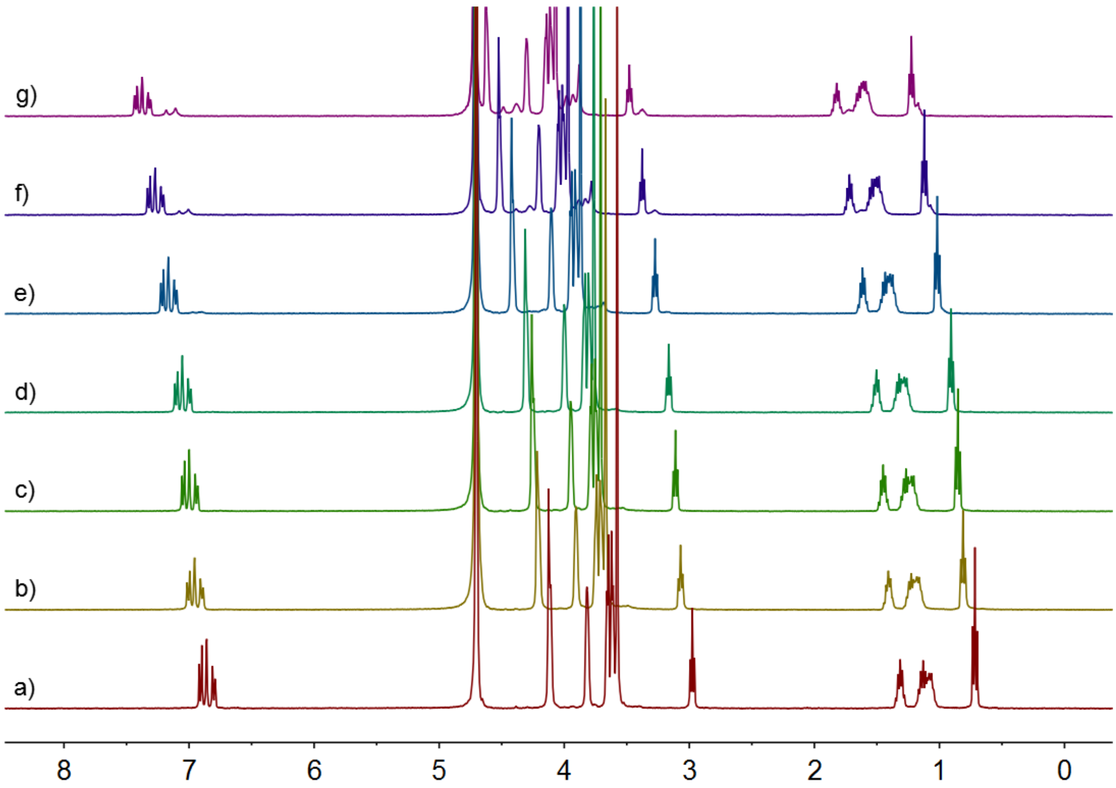

Figure S43: Temperature-dependent ^{1}H NMR spectra (400 MHz, D_2O) of **5d** (the concentration is 7.5 mg/mL). a) 25 ºC; b) 30 ºC; c) 35 ºC; d) 40 ºC; e) 50 ºC; f) 60 ºC; g) 70 ºC.

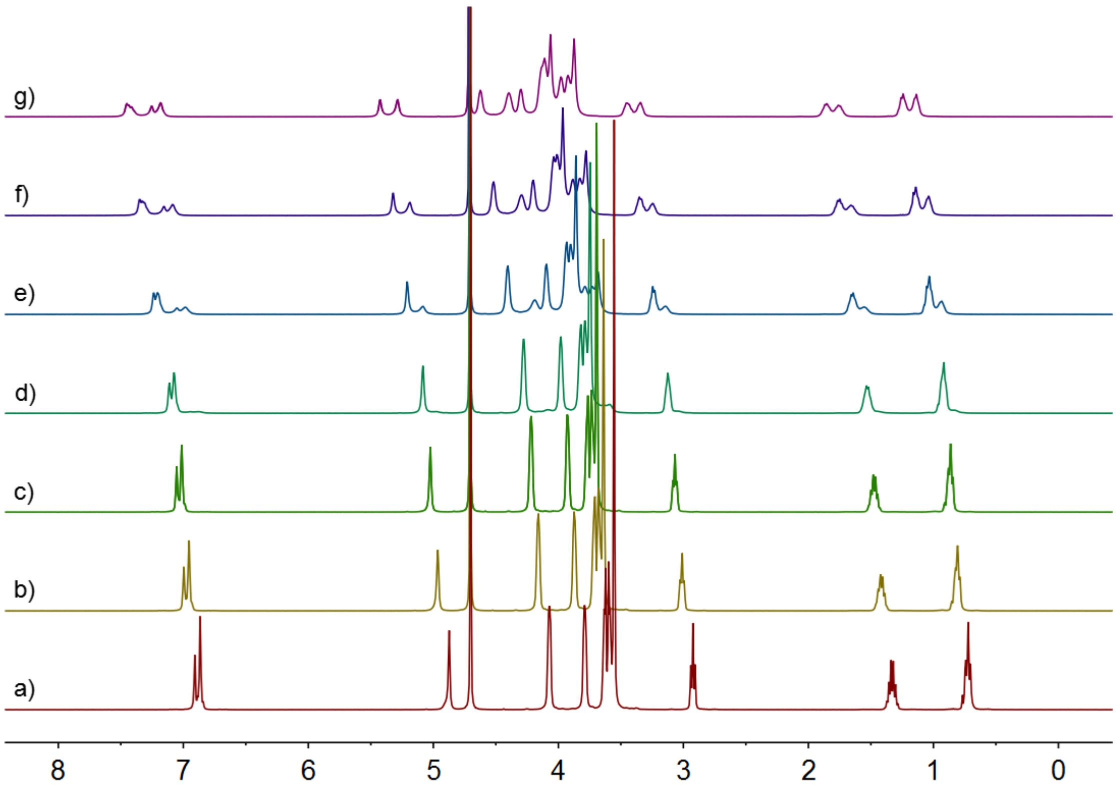

Figure S44: Temperature-dependent ^{1}H NMR spectra (400 MHz, D_2O) of **3a** (the concentration is 22 mg/mL). a) 25 ºC; b) 30 ºC; c) 35 ºC; d) 40 ºC; e) 50 ºC; f) 60 ºC; g) 70 ºC.

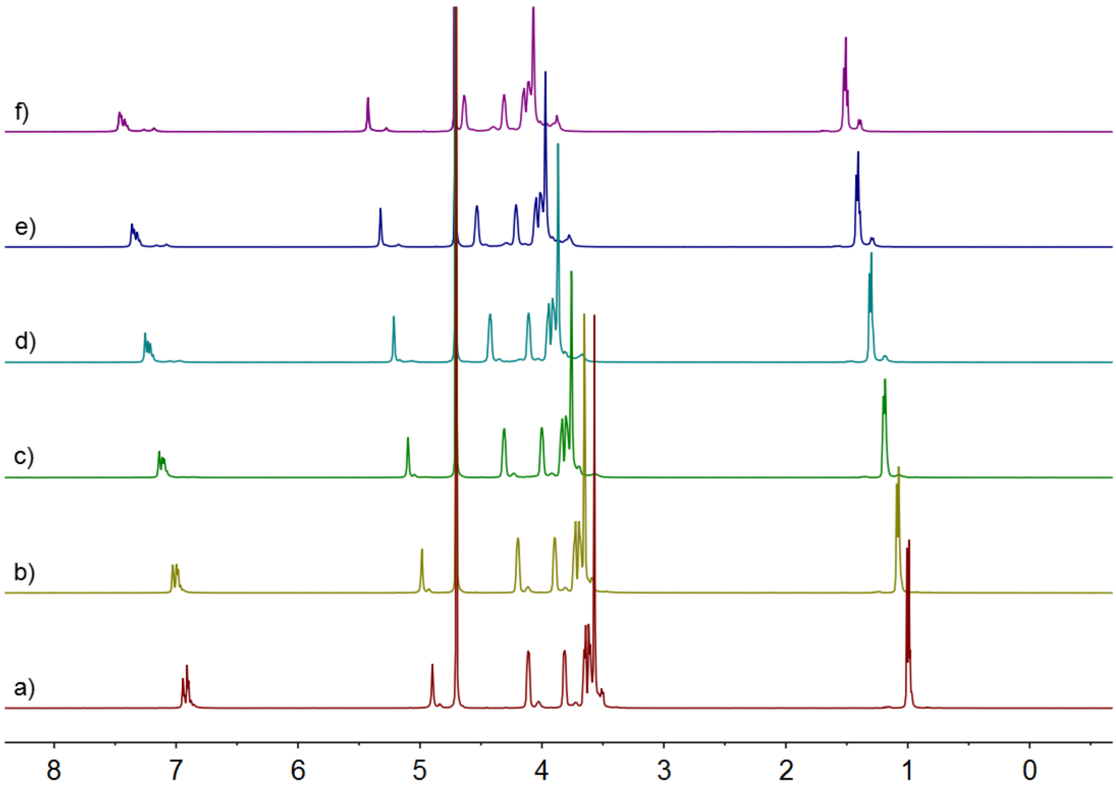

Figure S45: Temperature-dependent ^{1}H NMR spectra (400 MHz, D_2O) of **3b** (the concentration is 10 mg/mL). a) 25 ºC; b) 30 ºC; c) 40 ºC; d) 50 ºC; e) 60 ºC; f) 70 ºC.

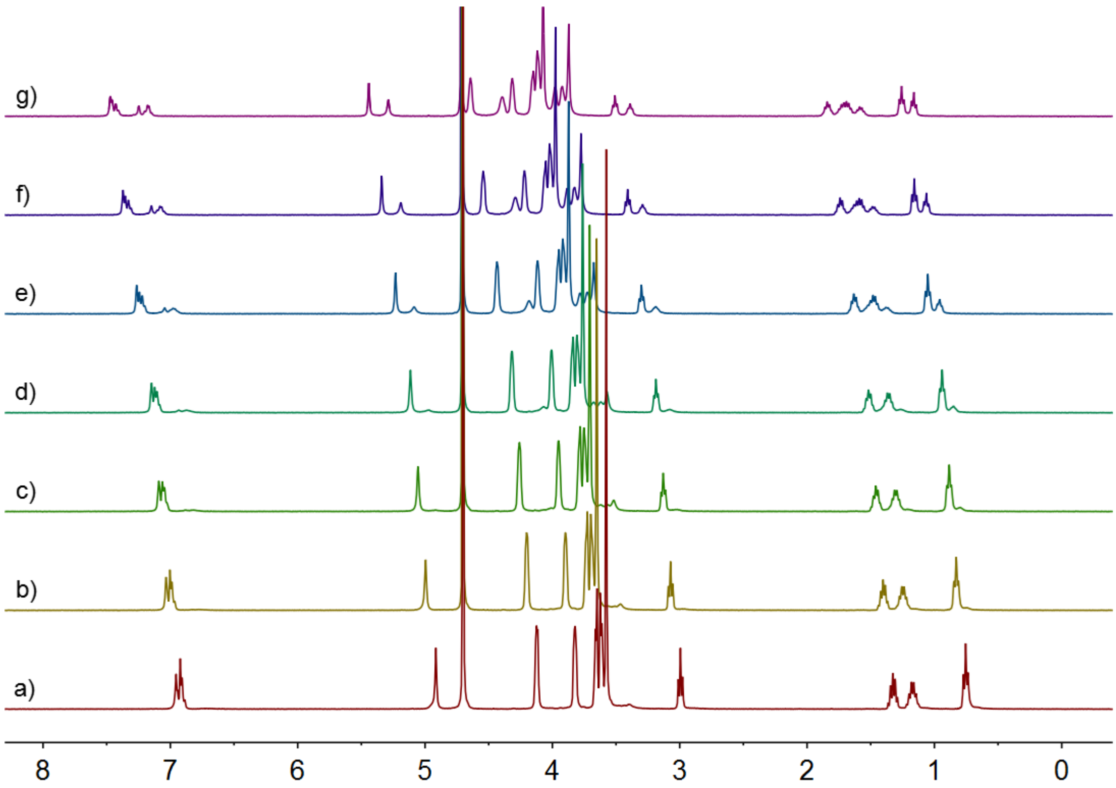

Figure S46: Temperature-dependent ^{1}H NMR spectra (400 MHz, D_2O) of **3c** (the concentration is 7 mg/mL). a) 25 ºC; b) 30 ºC; c) 35 ºC; d) 40 ºC; e) 50 ºC; f) 60 ºC; g) 70 ºC.

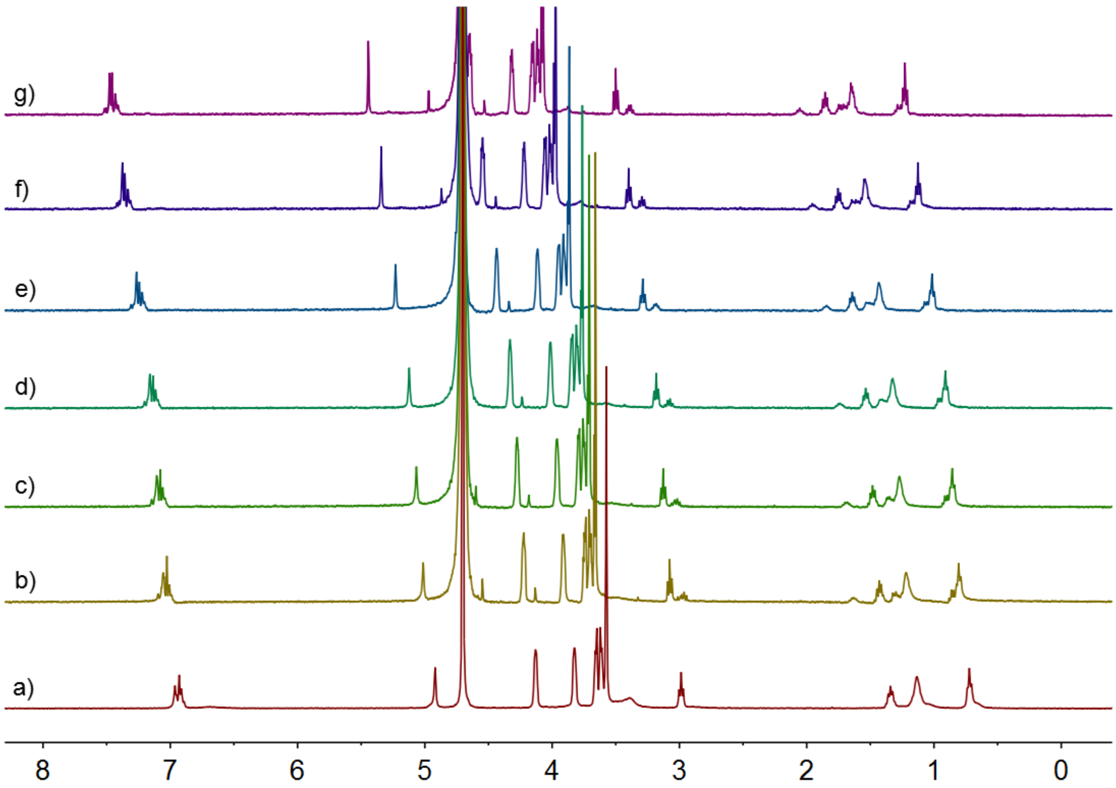

Figure S47: Temperature-dependent ^{1}H NMR spectra (400 MHz, D_2O) of **3d** (the concentration is 1 mg/mL). a) 25 ºC; b) 30 ºC; c) 35 ºC; d) 40 ºC; e) 50 ºC; f) 60 ºC; g) 70 ºC.

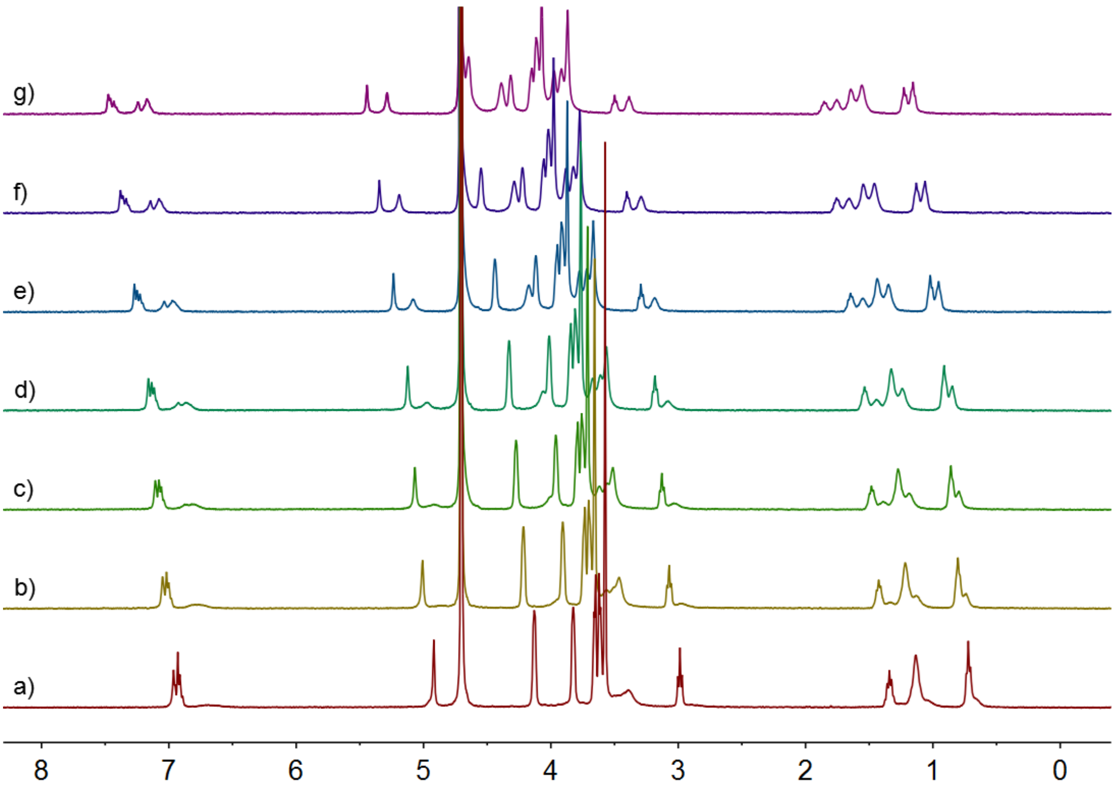

Figure S48: Temperature-dependent ^{1}H NMR spectra (400 MHz, D_2O) of **3d** (the concentration is 4 mg/mL). a) 25 ºC; b) 30 ºC; c) 35 ºC; d) 40 ºC; e) 50 ºC; f) 60 ºC; g) 70 ºC.

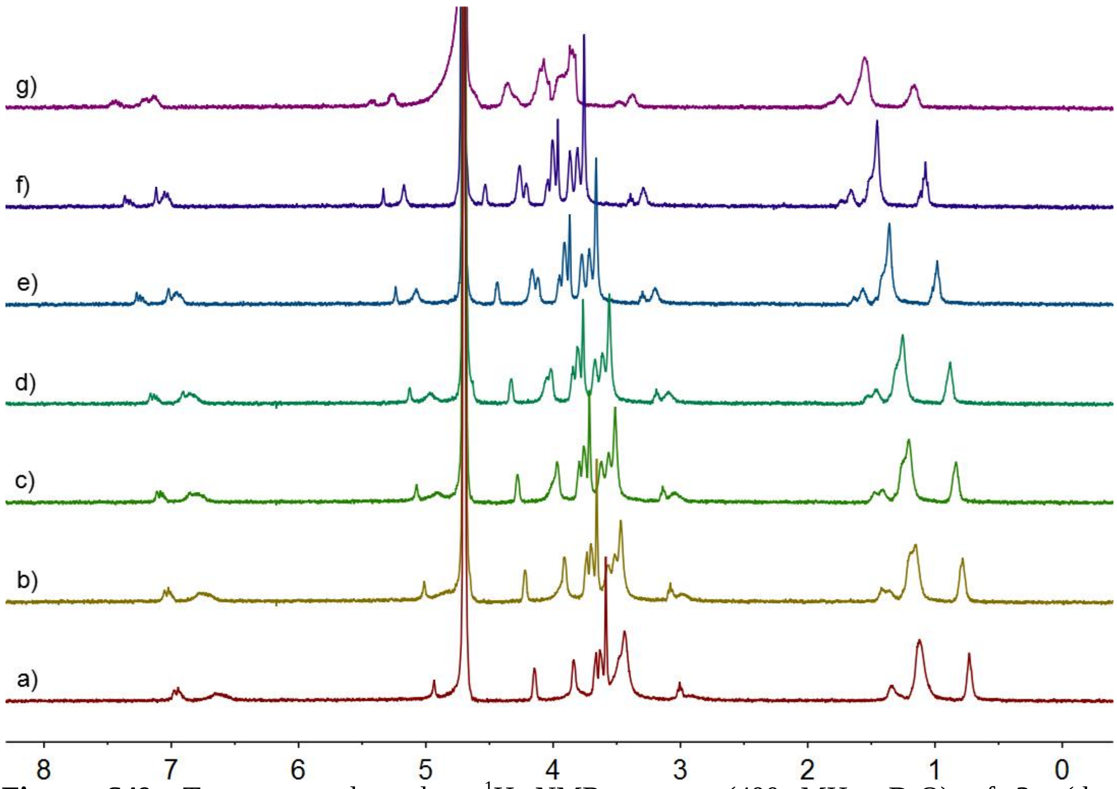

Figure S49: Temperature-dependent ^{1}H NMR spectra (400 MHz, D_2O) of **3e** (the concentration is 0.5 mg/mL). a) 25 ºC; b) 30 ºC; c) 35 ºC; d) 40 ºC; e) 50 ºC; f) 60 ºC; g) 70 ºC.

References

1. Ding, Y.; Wang, P.; Tian, Y.-K.; Tian, Y.-J.; Wang, F. *Chem. Commun*. **2013**, *49*, 5951–5953.

2. Qi, Z.; de Molina, P. M.; Jiang, W.; Wang, Q.; Nowosinski, K.; Schulz, A.; Gradzielski, M.; Schalley, C. A. *Chem. Sci.* **2012**, *3*, 2073–2082.
